# High‐Performance Transparent Solid Polymer Electrolyte Based on Copolymer of Deep Eutectic Electrolyte and Methyl Methacrylate for Electrochemical Devices

**DOI:** 10.1002/advs.76363

**Published:** 2026-06-28

**Authors:** Tingting Chen, Sijia Han, Likun Wang, Sainan Ma, Liping Zhang, Yong Liu, Gaorong Han

**Affiliations:** ^1^ School of Materials Science and Engineering Zhejiang University Hangzhou China; ^2^ Ningbo Global Innovation Center Zhejiang University Ningbo China

**Keywords:** electrochromic devices, lithium metal batteries, polymerizable deep eutectic electrolyte, solid polymer electrolyte

## Abstract

Polymerizable deep eutectic electrolytes (PDEEs) have emerged as next‐generation electrolytes for electrochemical devices, addressing the leakage risk of traditional DEEs and the safety concerns of flammable ionic liquid electrolytes. However, a significant challenge hindering further development is the scarcity of DEE‐based polymers capable of simultaneously balance the electrolyte conductivity, mechanical strength, and stability. Herein, a high‐performance poly(DEEs) was engineered by exploiting the synergistic effects of hydrogen bonding and ion‐dipole interactions via the incorporation of acrylamide (AM), succinonitrile, LiClO_4_, and methyl methacrylate (MMA). The solid polymer electrolytes were elaborately designed via in situ copolymerization, facilitating Li^+^ transport through hydrogen bond interaction with NH_2_ and C≡N groups, and utilizing the dual‐skeleton framework of AM and MMA to integrate electrochemical and mechanical properties. The optimized poly(DEE‐MMA) exhibits a high ionic conductivity of 1.43 mS cm^−1^, a superior Li^+^ transference number of 0.83, and excellent tensile strength of 17.8 MPa at 298 K, enabling the Li||LiFePO_4_ battery to deliver an impressive lifespan of 120 cycles with 83% capacity retention and the electrochromic device sustains 2000 coloration‐bleaching cycles with retaining 80% of the initial optical modulation. These findings provide a novel strategy for high‐performance solid polymer electrolytes and demonstrate potential applications in electrochemical devices.

## Graphical abstract

A copolymerization strategy using a polymerizable deep eutectic electrolyte with MMA forms efficient ion‐transport pathways while maintaining excellent transparency. The solid polymer electrolyte simultaneously achieves 17.8  MPa mechanical strength and 1.43  mS cm^−^^1^ ionic conductivity, enhancing long‐term cycling stability in Li|LFP batteries and electrochromic devices, providing an integrated approach for next‐generation multifunctional, high‐performance electrochemical systems.

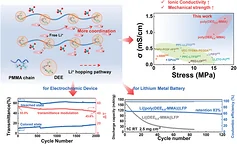

## 1 Introduction

Deep eutectic solvents (DESs) are composed of binary or ternary components via coordination between Brønsted or Lewis acids and bases, and exhibit significantly lower melting points as a result of strong hydrogen bonding and van der Waals interactions [1, 2]. This property enables the formation of room‐temperature liquids and provides a cost‐effective alternative to conventional ionic liquids with comparable electrochemical stability and environmentally friendly synthesis routes [3, 4, 5]. When employed as electrolytes in electrochemical devices DESs typically contain lithium salts (LiX, where X = PF_6_^−^, TFSI^−^, or ClO_4_^−^) in combination with Lewis bases such as succinonitrile (SN), urea, or N‐methylacetamide (NMAc), thereby forming deep eutectic electrolytes (DEEs) [6, 7]. DEEs have emerged as a promising candidate for advanced electrolytes due to their tunable hydrogen‐bond networks and nonflammable nature [8, 9]. However, the utilization of such high‐performance liquid electrolytes in energy devices that rely on electrochemical reactions still faces typical issues, such as leakage risk, gas generation, and a limited electrochemical window, which restrict their practical applications [10, 11]. Consequently, several efforts aim to achieve quasi‐solid or all‐solid states by combining DEEs with polymers to address these issues [12, 13].

Several strategies for preparing solid‐state electrolytes with DEEs have been developed through polymeric approaches that inherit the numerous advantages of DEEs, yet are constrained by inherent performance trade‐offs. Self‐polymerization of polymerizable deep eutectic electrolytes (PDEEs), characterized by polymerizable units within their hydrogen bond donors (HBDs) or hydrogen bond acceptors (HBAs), is utilized to synthesize poly(DEEs) [14]. This approach retains the ion transport advantages of DEE and enables in situ curing directly forming solid‐state electrolyte networks via photo‐ or thermally triggered polymerization [15]. However, this approach often results in compromised mechanical strength, which is insufficient to accommodate volume and stress changes between electrodes, thereby limiting long‐term cycling stability. Additionally, a strategy has been employed in which DEEs are encapsulated as plasticizers within a crosslinked polymer network with phase separation, in which rigid framework and ion transport channels are provided, enhancing both mechanical strength and ionic conductivity [16, 17]. Nevertheless, phase separation generally reduces the uniformity and controllability of internal structure distribution, thereby reducing the optical properties of PDEEs and imposing limitations on optical applications. Consequently, solid electrolytes that combine high conductivity, mechanical strength, and transparency are essential for multifunctional electrochemical devices, necessitating a synergistic co‐design approach between DEEs and the polymer matrix.

Solid polymer electrolytes (SPEs) have been studied extensively, with multiple structural designs that provide safety and tunable mechanical strength, rendering them essential components for practical electrochemical devices. Among the most widely studied polymer matrices, such as polyvinylidene fluoride (PVDF) [18], polyacrylonitrile (PAN) [19], and polyethylene oxide (PEO) [20], poly(methyl methacrylate) (PMMA) is particularly distinguished for applications requiring optical transparency (e.g., electrochromic devices, ECDs [21, 22]) due to its excellent visible‐light transmittance and outstanding mechanical properties [23]. These characteristics are attributed to the presence of polar carbonyl (C═O) groups, as well as a highly ordered, stereoregular configuration [24].

Here, we propose a transparent solid‐electrolyte strategy by copolymerizing ternary deep eutectic electrolytes with MMA to form poly(DEE‐MMA), in which multi‐branched PMMA serves as the backbone and deep eutectic units are copolymerized to optimize both electrochemical performance and mechanical properties. The strong eutectic interactions and hydrogen bonding of DEE, based on acrylamide (AM), succinonitrile (SN), and lithium perchlorate (LiClO_4_) (LiClO_4_ provides excellent stability and compatibility in various applications [25, 26]), enhance the dissociation efficiency of lithium salts in solid‐state electrodes while promoting the formation of polymer network structures that enable rapid ion transport. Ultimately, poly(DEE‐MMA) exhibits a high ionic conductivity (1.43 mS cm^−1^), a wide electrochemical window (4.6 V), and a lithium‐ion transference number of 0.83. Additionally, the strength of MMA and the elasticity of AM contribute to the mechanical stability, resulting in a high tensile strength of 17.8 MPa. With these properties, the poly(DEE‐MMA) serving as SPE in two typical electrochemical devices (Figure 1), a lithium metal battery (LMB) and an electrochromic device (ECD), demonstrates superior performance: Li||LiFePO_4_ full cells operating at 4.2 V exhibit a reversible capacity of 99.8 mAh g^−1^ and maintain 83% capacity after 120 cycles at 1.0 C, while a Prussian blue based semi‐ECD maintain 80% of initial optical modulation amplitude after 2000 cycles. These findings highlight the effectiveness of this approach in developing novel poly(DEE‐MMA) electrolytes with superior performance, establishing a valuable foundation for future research on multifunctional advanced electrochemical devices.

**FIGURE 1:**
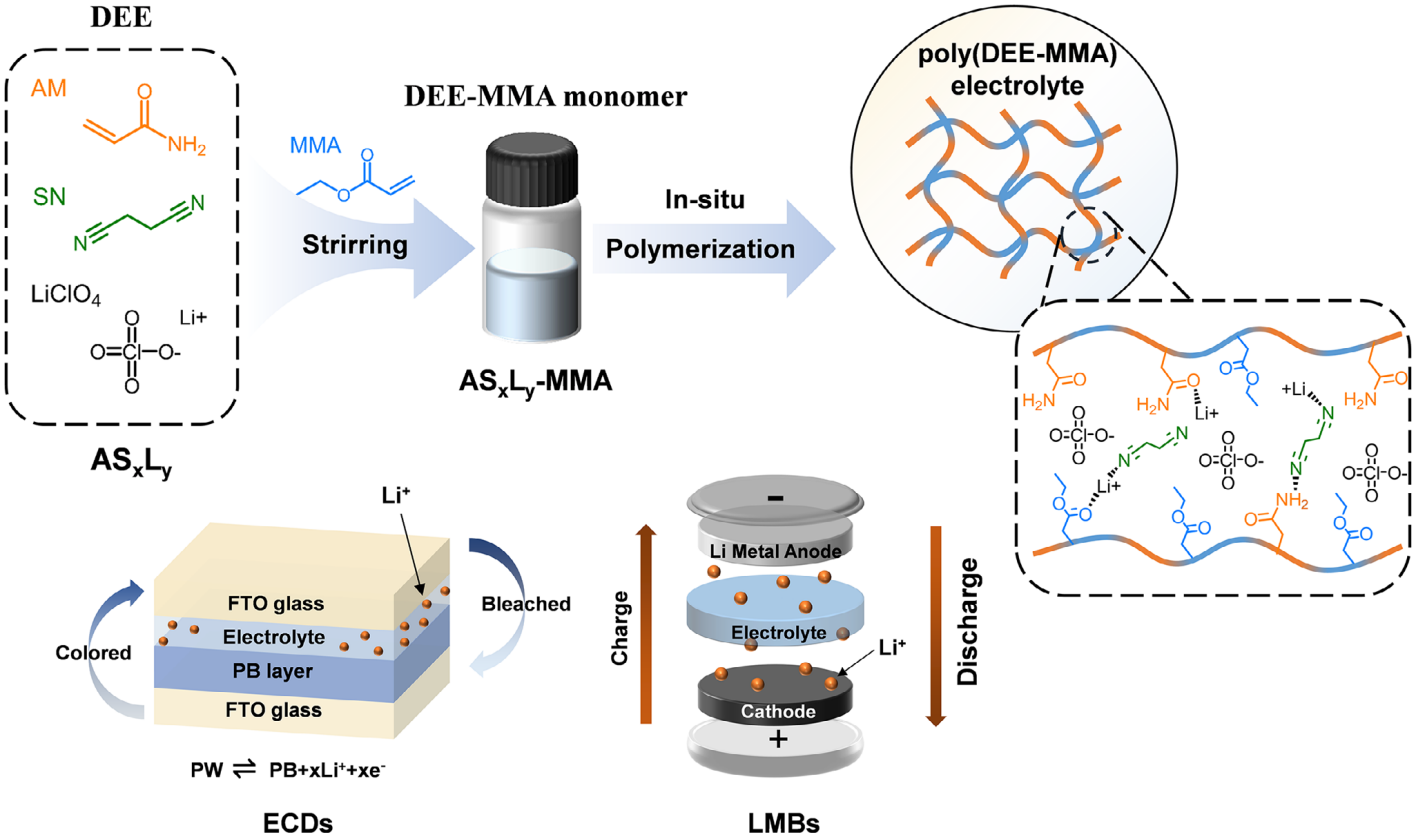
Schematic diagram of the design strategy and the device structure of ECDs and LMBs.

## 2 Results and Discussion

### 2.1 Characterization and Formation Mechanism of DEE

Ternary DEEs were successfully prepared by mixing AM, SN, and LiClO_4_ in various molar ratios (Figure S1), designated as AS_x_L_y_ (see experimental section for details, x = 2, 3, 4, 8, and y = 0.2, 0.4, 0.6, 0.8, 1.2). The prepared AS_x_L_y_ exhibits extended eutectic behavior, forming a stable liquid phase at room temperature (T < 25°C) within specific molar ratio ranges (Figure S2), with melting points substantially lower than those of the individual components (SN: 142.5°C; AM: 110.6°C; LiClO_4_: 236°C), indicating the successful formation of the ternary DEE systems [27, 28]. More than 50 solution samples were prepared to construct the ternary phase diagram, as shown in Figure 2a, to further delineate the boundary conditions of the eutectic region. Thermodynamic analysis of the selected samples, AS_4_L_y_ and AS_8_L_y_, shows that the melting point ranges from −12°C to 14°C, decreasing with increasing lithium salt content and increases with higher SN content (Figure 2b and Figure S3), suggesting that the deep eutectic region favors higher lithium salt. Effective hydrogen bonding interactions within the DEE systems also impart excellent flame‐retardant properties to the eutectic electrolytes (Figure S4), further emphasizing their intrinsic safety advantage compared with conventional commercial electrolytes.

**FIGURE 2:**
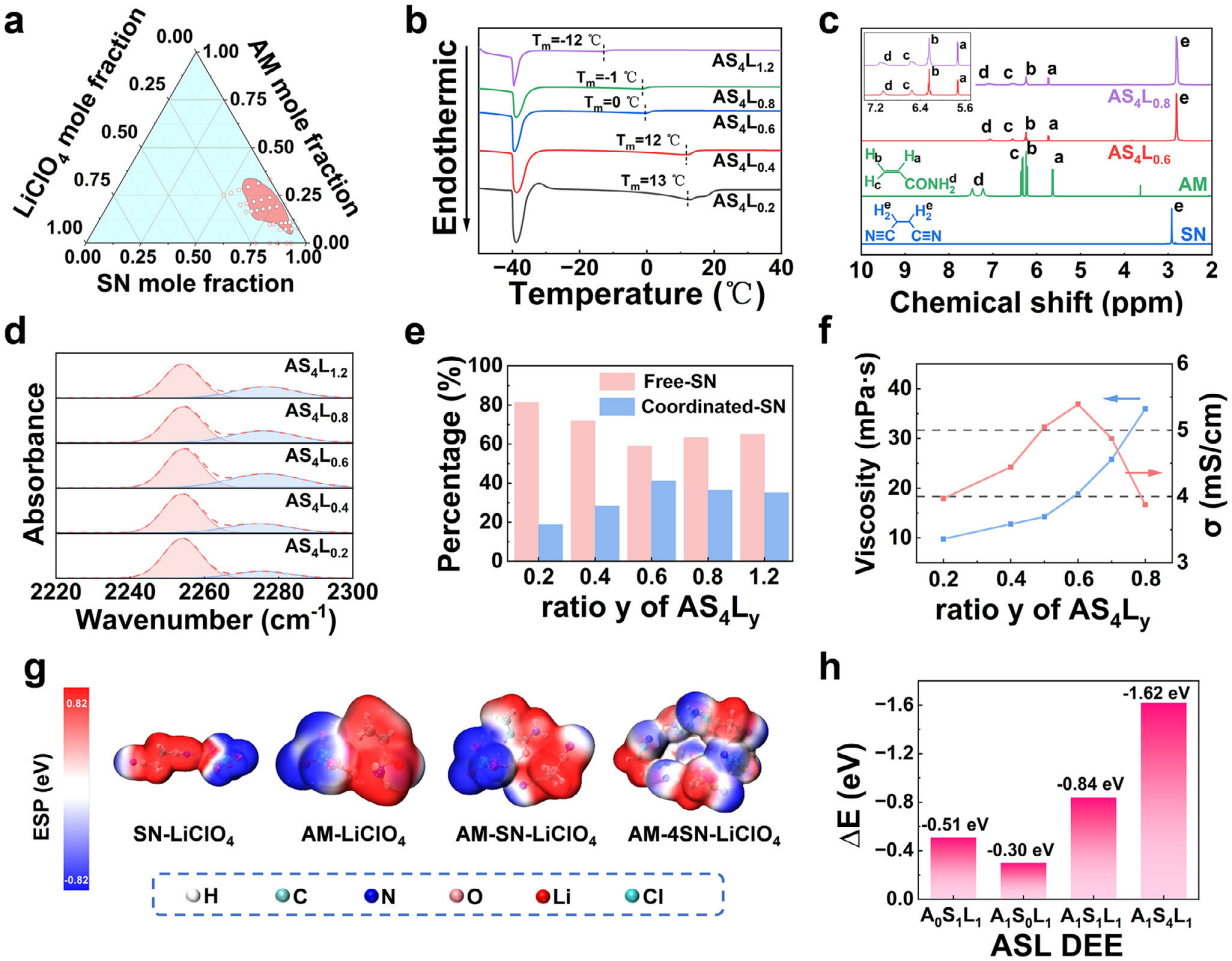
(a) Ternary phase diagram of the solid‐liquid system drawn from experimental data at 25°C, with the liquid phase indicated in the pink region; (b) DSC spectra of AS_4_L_y_ (T_m_: melting temperature); (c) ^1^H NMR spectra of AM, SN, and two samples of ASL; (d) FTIR spectra of C≡N peaks and (e) free‐SN and coordinated‐SN distributions obtained from the FTIR spectra in AS_4_L_y_ DEE; (f) Ionic conductivity and viscosity of AS_4_L_y_ DEE; (g) Calculated ESP distribution; and (h) the binding energy of SN, AM, and LiClO_4_ ternary DEE with various ratios.

To gain further insight into the formation mechanism of the ternary DEE system, nuclear magnetic resonance (NMR) and Fourier transform infrared (FTIR) spectroscopy were performed. As shown in Figure 2c, the ^1^H NMR spectra display the characteristic chemical shifts of each component, indicating that no chemical reaction occurred during DEE preparation. A progressive downfield shift of the AM amide proton (δ = 7.22 ppm) exhibits a shift exceeding 0.14 ppm resulting from hydrogen bonding with the SN nitrile group. Additionally, the chemical environment of the SN active hydrogen (δ = 2.92 ppm) is slightly affected by changes in the nitrile environment, leading to a shift of less than 0.1 ppm (Table S1). These observations confirm a deshielding effect, corresponding to reduced electron density around the hydrogen atoms, and indicate the formation of numerous hydrogen bonds between the components [29, 30]. This result is further supported by FTIR spectroscopy, in which the amide group signal in DEE exhibits a blueshift compared to pure AM (Figure S5). FTIR analysis was conducted to compare the ion‐dipole interactions and solvation structures within DEE as a function of the LiClO_4_‐to‐AM molar ratio (0.2:1, 0.4:1, 0.6:1, 0.8:1, and 1.2:1). Compared to pure SN, the FTIR spectrum of DEE shows a new peak near 2275 cm^−1^, attributed to interaction with Li^+^ ions. As shown in Figure 2d, the peaks at 2253 and 2276 cm^−1^ correspond to free SN and Li^+^‐coordinated SN, respectively. The relative intensity of the peak at 2279 cm^−1^ initially increases with rising LiClO_4_ content (from AS_4_L_0.2_ to AS_4_L_0.6_), indicating a higher degree of SN coordination to Li^+^ in AS_4_L_0.6_ compared to the other DEEs. To quantify the degree of coordination, the area ratios of Li^+^ coordinated C≡N groups and free SN molecules are estimated as shown in Figure 2e [31], with AS_4_L_0.6_ exhibiting the highest coordinated nitrile area ratio of approximately 40%, reflecting that effective ion‐dipole interactions establish a favorable environment for Li^+^ dissociation. Meanwhile, as shown in Figure 2f, ionic conductivity initially increases and subsequently decreases with increasing LiClO_4_ content, reaching a maximum of 5.39 mS cm^−1^ at AS_4_L_0.6_, consistent with the dissociation trend. The viscosity of DEEs increased monoclinically with lithium salt concentration. When the LiClO_4_ content exceeded AS_4_L_0.6_, a pronounced increase in viscosity is observed, which, combined with dissociation effects, leads to a reduced ionic conductivity. This effect was further verified in additional samples, where mutual coordination interaction between SN and Li^+^ were found to enhance ionic conductivity (Figures S6 and S7). This interaction follows the same trend as the ionic conductivity with varying x‐ratios in AS_x_L_0.6_. Thus, by regulating the ternary eutectic ratio, modulating the hydrogen‐bond interactions among nitrile, amide groups, and Li^+^, as well as controlling the dissociation and transport of Li^+^ by the nitrile group, tunable ionic conductivity is achieved in the ternary DEE samples. AM and SN modulate both the Li^+^ coordination geometry and the hydrogen‐bonding environment, thereby enhancing ion dissociation, improving electrolyte fluidity, and stabilizing the eutectic structure.

To better understand the mechanism, DFT calculations were carried out. The electrostatic potential (ESP) was calculated to identify the electronegative and electropositive atoms of LiClO_4_, AM, and SN, which are primarily responsible for molecular coordination, as shown in Figure 2g. For simplicity, the smallest integer ratio of the ASL system was employed for modeling and calculation. The ESP results indicate that the C≡N group in SN and the C═O group in AM are highly electronegative functional groups, exerting a strong electrostatic influence on Li ions. Additionally, A_1_S_4_L_1_ exhibits the lowest ESP, which is relatively uniform across the molecule, suggesting a reduced anion effect on Li^+^ diffusion [32]. To quantitatively compare coordination across different ratios, binding energies were calculated shown in Figure 2h. The binding energy of A_1_S_1_L_1_ (AM: SN: LiClO_4_ = 1:1:1) is −0.84 eV, higher than those of A_0_S_1_L_1_ (AM:SN:LiClO_4_ = 0:1:1, −0.51 eV) and A_1_S_0_L_1_ (AM:SN:LiClO_4_ = 1:0:1, −0.30 eV), reflecting stronger dissociation interactions between Li^+^ and the hydrogen‐bonding groups of SN and AM. Furthermore, when the SN to AM ratio reaches 4:1, the ASL system exhibits an even higher binding energy of −1.62 eV, indicating that AS_4_L_y_ samples display enhanced interactions. The increase in binding energy, together with the decrease in interatomic distance and dipole moment, thereby promoting Li^+^ solvation [33]. Enhanced solvation facilitates more uniform Li^+^ flux and improved overall Li^+^ conductivity.

Experimental characterization and theoretical calculations collectively confirm that a deep eutectic system with a specific stoichiometric ratio is capable of improving the binding energy between components by optimizing hydrogen‐bond dissociation and complexation. This promotes dissociation and low‐energy‐barrier transport of lithium salts, thereby enhancing the electrochemical performance of the DEE system. These findings indicate that the optimal DEE composition, with an AM:SN:LiClO_4_ ratio of 1:4:0.6, provides the highest SN coordination within the DEE, which was subsequently employed for in situ copolymerization of solid‐state electrolytes.

### 2.2 Synthesis and Characterization of Poly(DEE‐MMA)

The transparent precursor solution containing AS_4_L_0.6_ and MMA was thermally copolymerized to form poly(DEE‐MMA), a solid polymer electrolyte (Figure S8). The x‐ray diffraction (XRD) patterns of samples with various compositions showed only broad humps without sharp peaks, indicating an amorphous polymeric structure, as shown in Figure 3a. The disappearance of the characteristic C═C peaks at 1608 cm^−1^ and 1637 cm^−1^ in the infrared spectra confirms the completion of polymerization in poly(DEE‐MMA), as shown in Figure 3b. This observation is further supported by the absence of peaks corresponding to C═C bonds in the 100—160 ppm range of the ^13^C NMR spectrum (Figure S9).

**FIGURE 3:**
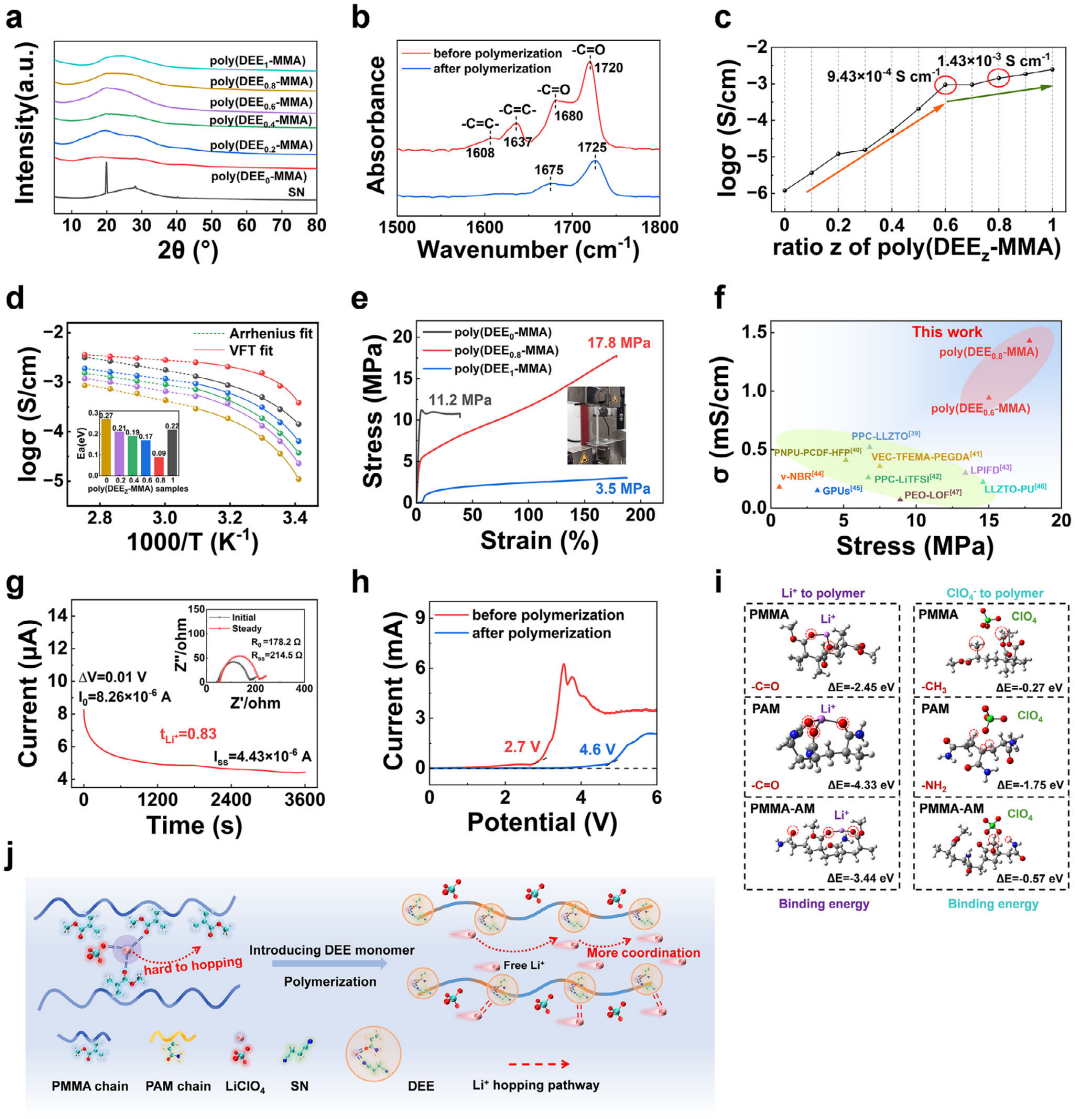
(a) XRD profiles of poly(DEE_Z_‐MMA); (b) FTIR spectra of poly(DEE‐MMA) electrolyte before and after polymerization; (c) Ionic conductivity of poly(DEE_Z_‐MMA) vs. DEE mass fraction z; (d) Temperature‐dependent ionic conductivity of poly(DEE_Z_‐MMA) (inset shows E_a_ of different samples); (e) Stress‐strain curves for different electrolyte tested at a rate of 20 mm min^−1^ and a digital photo of the electronic universal tensile testing machine; (f) Comparison of mechanical strength and ionic conductivity with reported literature [39, 40, 41, 42, 43, 44, 45, 46, 47]; (g) Chronoamperometry profile of Li|poly(DEE_0.8_‐MMA)|Li cells under a 10 mV polarization voltage with EIS spectra before and after polarization in the inset; (h) LSV curves of the poly(DEE_0.8_‐MMA) and DEE_0.8_‐MMA electrolytes at a scan rate of 10 mV s^−1^; (i) The binding energies and corresponding adsorption configurations of Li^+^ and ClO_4_^−^ to PMMA, PAM and PMMA‐AM chains; (j) Schematic illustration of the transport structure transport structure in poly(DEE‐MMA) after copolymerization.

The ionic conductivity of poly(DEE‐MMA) as a function of DEE mass fraction is shown in Figure 3c, exhibiting a merely monotonic increase. The ionic conductivity reaches 0.93 mS cm^−1^ at a DEE ratio of 0.6 from 1.21 × 10^−3 ^mS cm^−1^ (0 wt.% DEE). After that, an inflection point in the conductivity growth rate emerges, which is a slight increase to 1.43 mS cm^−1^ when the DEE ratio further rises to 0.8, potentially indicating different mechanisms of ionic conductivity enhancement (Figure S10). Initially, the introduced DEE rapidly establishes percolative transport pathways within the MMA‐based network, thereby accelerating Li^+^ transport and leading to a sharp rise in conductivity. After the DEE ratio exceeds approximately 0.6, these pathways become nearly saturated, and the contribution of subsequent additions to ion conduction diminishes, resulting in a slower growth rate. Figure 3d shows the temperature dependence of the ionic conductivity for poly(DEE‐MMA), as determined from EIS measurements. At lower temperatures (below the glass transition region), the conductivity is described by the Vogel‐Tamman‐Fulcher (VFT) behavior, in which Li^+^ migration is coupled with the structural relaxation and segmental mobility of the polymer matrix. In the higher temperature region, Arrhenius behavior is observed, indicating that ion transport is dominated by fast‐conduction pathways facilitated by small molecules and polymer chain functional groups following sufficient segmental relaxation [34, 35] (Figure S11, Equations S1 and S2, and Table S2). E_a_ as calculated by Arrhenius is observed to decrease systematically with increasing DEE content, with poly(DEE_0.8_‐MMA) exhibiting the lowest energy barrier of 0.11 eV, which facilitates rapid ion transport. This activation energy is significantly lower than that reported for SPEs such as PEO (0.65 eV), indicating accelerated Li^+^ transport kinetics for the poly(DEE‐MMA) system [36, 37].

In poly(DEE‐MMA), the methyl methacrylate groups serve as rigid units, while the amide groups act as flexible units, together endowing the copolymer with outstanding mechanical properties [38]. The mechanical properties of SPEs were assessed using a universal tensile testing machine, and the resulting tensile stress‐strain curves for various electrolyte samples (Figure 3e and Figure S12). As expected, the AM‐MMA copolymer achieves a well‐balanced mechanical performance, combining high tensile strength (11.2 MPa) with excellent flexibility (160% elongation) — substantially outperforming the MMA‐only sample in ductility (46%) and the AM‐only sample in strength (3.5 MPa). This significant mechanical improvement is attributed to the dual‐skeleton effect, which synergistically combines the structural reinforcement provided by the rigid PMMA network with the elastic enhancement from the flexible AM network. Such excellent elasticity enables poly(DEE‐MMA) to accommodate the volume expansion of the electrode during electrochemical reactions. The novel poly(DEE_0.8_‐MMA) polymer electrolyte overcomes the classic trade‐off between ionic conductivity and mechanical strength, as shown in Figure 3f [39, 40, 41, 42, 43, 44, 45, 46, 47]. These findings demonstrate the tunability of material properties achieved through the copolymerization of DEE and MMA for SPE preparation, effectively validating the structural advantages of our copolymer design.

The lithium‐ion transference number (t_Li+_) is a pivotal parameter for electrolytes, as a low t_Li+_ can lead to increased electrode polarization and Li dendrite formation. Figure 3g shows the time‐dependent response of direct current polarization for the poly(DEE_0.8_‐MMA) electrolyte, with the inset displaying impedance spectra before and after chronoamperometry. The poly(DEE_0.8_‐MMA) electrolyte achieves a high t_Li+_ of 0.83, which is facilitated by the synergistic interplay between carbonyl (C = O) groups and DEE‐induced hydrogen‐bonding interactions. This molecular environment promotes effective salt dissociation, accelerates selective cation transport, and simultaneously suppresses anion mobility. The obtained t_Li+_ value substantially surpasses those of conventional systems — 0.52 for 1 m LiClO_4_ in PC and 0.18 for the unmodified poly(DEE_0_‐MMA) (Figure S13) — underscoring the effectiveness of the deep eutectic design in modulating ion transport dynamics. Such a high t_Li+_ is primarily attributed to inhibited anion migration within the polymer matrix, reducing concentration polarization and subsequent side reactions in lithium metal batteries [48]. Additionally, linear sweep voltammetry (LSV) was employed to assess the electrochemical stability of the electrolyte samples, as shown in Figure 3h. The oxidation potential of the DEE_0.8_‐MMA monomer electrolyte was 2.7 V vs. SS/SS. After copolymerization, the electrochemical stability of poly(DEE_0.8_‐MMA) was enhanced to 4.6 V vs. SS/SS, which is attributed to the polymer chains providing high resistance to electrochemical oxidation [49].

To explore ion‐transporting mechanisms, the adsorption energies of Li^+^ and ClO_4_^−^ on different polymer chain segments were calculated using DFT. As shown in Figure 3i, all chain segments show significantly stronger interactions with Li^+^ than with ClO_4_^−^, preferentially separating Li^+^‐ClO_4_^−^ ion pairs and providing selective binding sites for Li^+^. Interaction energies of Li^+^ with carbonyl groups in PMMA and PAM (polymerization product of AM) were approximately −2.45 and −4.33 eV, respectively, compared to −3.44 eV in the PMMA‐AM copolymer segments. The copolymer segments exhibit moderate binding energy toward Li^+^, preventing either excessive binding that restricts Li^+^ ion mobility or insufficient interaction that prevents dissociation from LiClO_4_. These results suggest low ionic activation energy and high Li^+^ mobility in the modified polymer electrolyte. The mechanism of rapid Li^+^ transport through deep eutectic groups within the copolymer block structure is schematically illustrated in Figure 3j. Flexible motion of the copolymer network segments facilitates coordination‐dissociation‐recoordination of Li^+^ across various sites, thereby enabling rapid Li^+^ migration pathways. Conversely, interaction between chain segment groups and ClO_4_^−^ restricts free diffusion of this bulky anion, reducing its effective migration and minimizing undesirable reactions at the anion‐electrode interface.

### 2.3 Electrochemical Performance of LMB

The Li||Li cell assembled poly(DEE_0.8_‐MMA) exhibited stable Li plating and stripping behavior at 0.1 mA cm^−2^, with an average overpotential of 48.3 mV for over 250 h, as shown in Figure 4a. This performance surpasses that observed for unpolymerized DEE‐MMA monomer electrolytes, which fail after 100 h. Similarly, the sample without DEE modification fails after 120 h (Figure S14). The Li foil using poly(DEE_0.8_‐MMA) remains flat after cycling (Figure S15). However, the application of a DEE_0.8_‐MMA electrolyte resulted in the formation of a conspicuously thick black layer on the surfaces of the Li foil. The chemical composition of the SEI was then detected by XPS. N 1s spectra can be divided into peaks assigned to C≡N (∼399.8 eV) and Li_3_N (∼398.5 eV), which are derived from the SN and AM products (Figure S16). Notably, after 250 cycles, the formed SEI layer exhibits a relatively high content of Li_3_N. As a fast ionic conductor, the presence of Li_3_N effectively mitigates concentration polarization and contributes to the stable cycling behavior of the lithium metal anode [50]. To further investigate the interfacial kinetics of lithium metal during electrochemical cycling, EIS measurements were performed on Li||Li symmetric cell every 50 cycles. EIS measurements and equivalent circuit [51] of Li||Li cells are shown in Figure 4b. The R_ct_ during cycling (160–200 Ω) is substantially reduced compared with the initial state (307 Ω), reflecting a marked decrease in the interfacial charge transfer barrier (see Figures S17 and S18 and Table S3). This reduction is due to the reduction of interface resistance, which shows that our in situ copolymerization of DEE and MMA strategy can effectively improve the interface performance [52].

**FIGURE 4:**
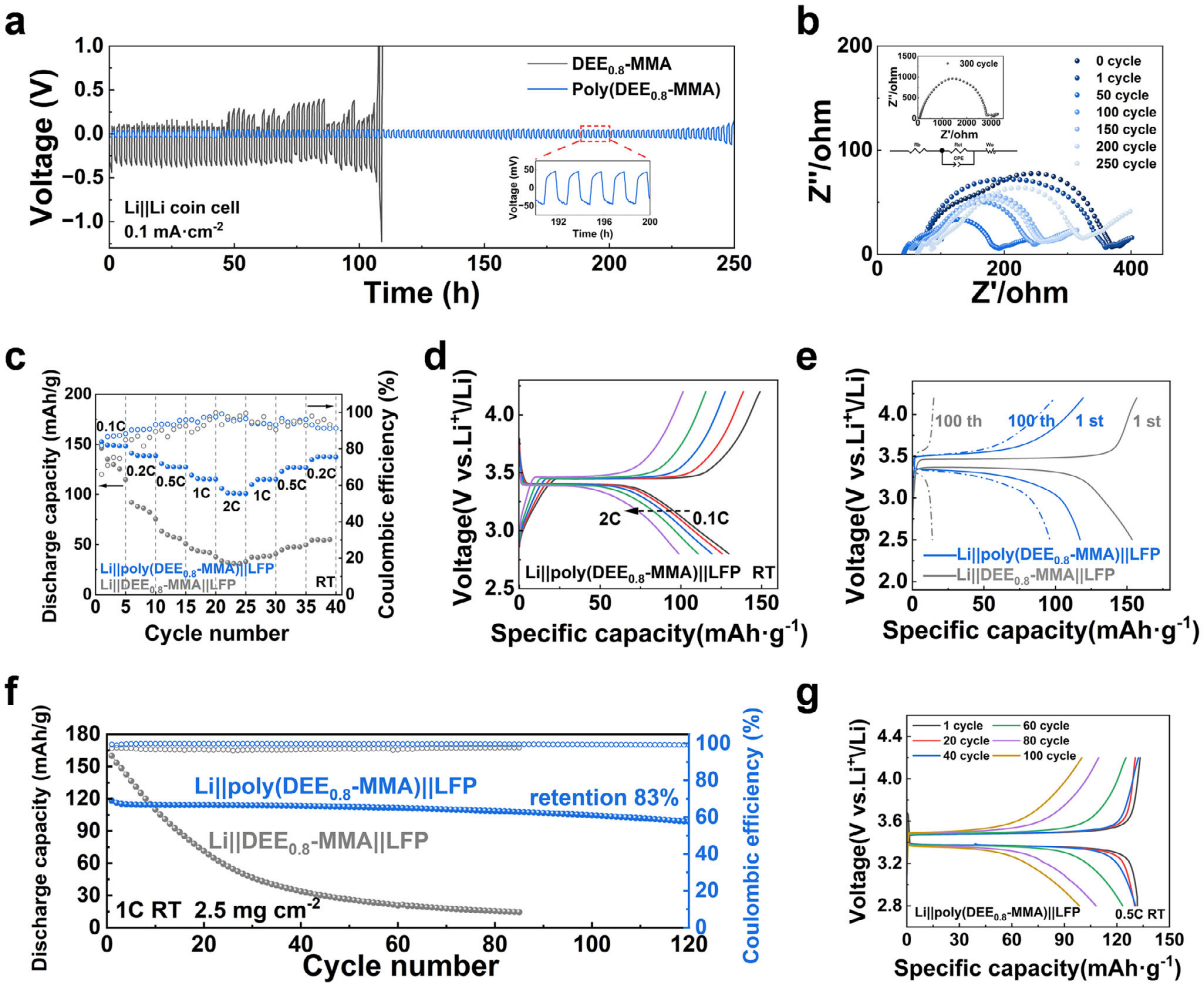
(a) Galvanostatic cycling curve of a Li symmetric cell at a current density of 0.1 mA cm^−2^; (b) EIS curve of the Li||Li symmetric battery at every 50 cycles; (c) Rate performance comparison of Li|poly(DEE_0.8_‐MMA)|LFP and Li|DEE_0.8_‐MMA|LFP cells from 2.5 to 4.2 V at room temperature; (d) Typical charge and discharge profiles of the Li||poly(DEE_0.8_‐MMA)||LFP cells at 0.1 C – 2.0 C; (e) Selected charging/discharging curves of cells; (f) Cycling performance of Li||poly(DEE_0.8_‐MMA)||LFP and Li||DEE_0.8_‐MMA||LFP cells at RT and 1C; (g) Selected charging/discharging curves of cells at RT and 0.5 C.

To evaluate the practical applicability of poly(DEE_0.8_‐MMA) electrolyte in lithium metal batteries (LMBs), a series of Li||LiFePO_4_ (LFP) cells was constructed using either the polymerized poly(DEE_0.8_‐MMA) or the corresponding DEE_0.8_‐MMA monomer as the electrolyte. As shown in Figure 4c, the Li|poly(DEE_0.8_‐MMA)|LFP battery exhibited highly reversible discharge behavior. At current densities of 0.1C, 0.2C, 0.5C, 1C, and 2C, the battery delivered discharge capacities of 152.5, 141.1, 130.7, 119.3, and 105.9 mAh g^−1^, respectively. Even at 2 C, the discharge specific capacity remained above 100 mAh g^−1^, exceeding that of cells using the DEE_0.8_‐MMA monomer electrolyte. Furthermore, the Li|poly(DEE_0.8_‐MMA)|LFP battery also exhibited stable charge–discharge profiles across various current rates within a voltage range of 2.5 to 4.2 V, as shown in Figure 4d.

As shown in Figure 4e,f, the cell equipped with poly(DEE_0.8_‐MMA) delivered an initial discharge capacity of 118.24 mAh g^−^^1^ and maintained 99.35 mAh g^−^^1^ after 120 cycles at 1 C, demonstrating excellent long‐term cycling stability with a capacity retention of 83%. In comparison, cells using the unpolymerized DEE_0.8_‐MMA monomer electrolyte showed rapid capacity fading, with substantial loss after only 30 cycles. The poly(DEE_0.8_‐MMA)‐based cell also exhibited a slightly higher average coulombic efficiency than the monomer precursor, indicating improved electrochemical reversibility and enhanced interfacial stability. Additional assessments under varied operational conditions, including cycling at 0.5 C under room temperature and at 1 C under an elevated temperature of 60°C, consistently corroborated the robust cycling performance and thermal stability of poly(DEE_0.8_‐MMA) electrolyte (Figure 4g and Figure S19). LFP batteries employing the poly(DEE_0.8_‐MMA) electrolyte exhibited remarkable durability, maintaining 94% of the initial capacity after 1000 cycles at a high rate of 5 C (Figure S20). In contrast, LFP batteries using DEE_0.8_‐MMA or commercial electrolytes exhibited poor cycling stability. This phenomenon may be attributed to the parasitic reaction between metallic lithium and the electrolyte, which leads to the continuous formation of an unstable solid electrolyte interphase that consumes both active lithium and the electrolyte. Compared with previous literature (Table S4), the deep eutectic‐modified copolymer solid polymer electrolyte developed here exhibits outstanding cycling performance in lithium metal batteries, demonstrating strong potential as a novel strategy for future deep eutectic modification approaches.

### 2.4 Electrochromic Performance of ECD

Electrochromic devices (ECDs) offer high controllability, rapid optical response, extended operational lifespan, and versatility across multiple service scenarios. Moreover, EC technology holds immense application potential in smart windows, anti‐glare rearview mirrors, smart displays, and information encryption [53]. The outstanding optical transparency of poly(DEE‐MMA) prompted us to explore its potential applications in optical devices for ECDs. Transmittance curves for samples of each composition were measured from 400 to 1600 nm using a spectrophotometer, as shown in Figure 5a. The poly(DEE_1_‐MMA) sample exhibits uneven and rough surfaces, enhancing light scattering and resulting in a macroscopic decrease in transmittance. The remaining samples, optimized with MMA copolymers, exhibit suppressed localized phase variations and a smooth surface. All samples are transparent, with transmittance of ∼90% in the visible range (400 ∼ 700 nm), and a photograph of a representative poly(DEE_0.8_‐MMA) is shown in the inset of Figure 5a. A quasi‐device with two blank FTO electrodes filled with poly(DEE_0.8_‐MMA) exhibits transmittance comparable to that of the PC‐LiClO_4_ liquid electrolyte (Figure S21), thereby meeting the visual requirements for smart window applications [54, 55]. Additionally, it is flexible and self‐supporting, demonstrating potential for future use in flexible devices [56]. The lap shear strength of poly(DEE_0.8_‐MMA) was measured using a universal testing machine to evaluate the mechanical adhesive strength between two glass panels, as shown in Figure 5b, with a digital photo inset in accordance with ASTM International standard test method D1002 [57]. The poly(DEE_0.8_‐MMA) exhibited a tensile strength of 1.23 MPa, which is 3.5 times higher than that of the reported DEE‐based gel electrolyte (0.35 MPa) [58], demonstrating strong adhesive properties.

**FIGURE 5:**
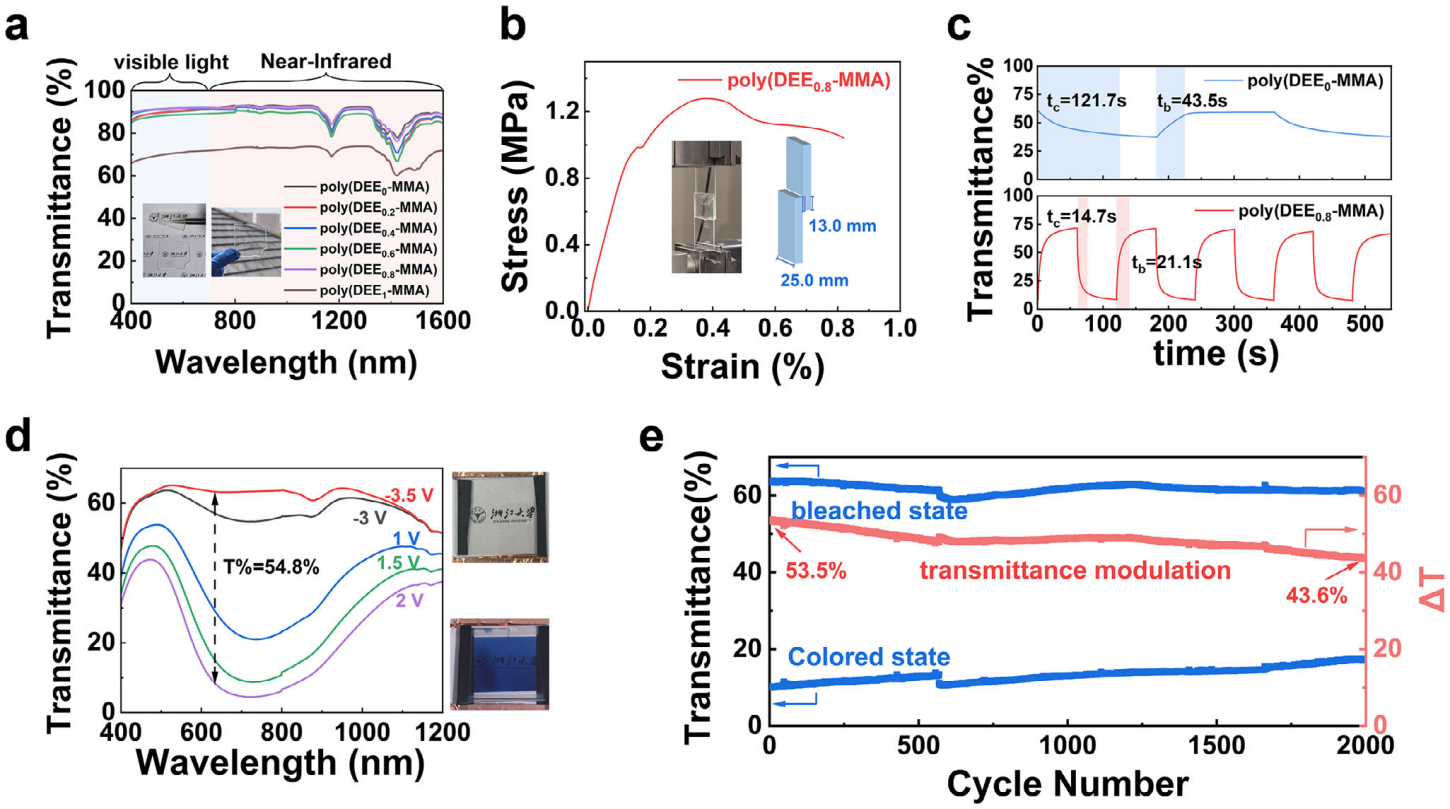
(a) Transmittance spectra and a photograph of the poly(DEE_z_‐MMA) samples; (b) Stress‐strain curves acquired during the lap‐shear‐strength tests; (c) In situ transmittance of the semi‐ECD with poly(DEE_0_‐MMA) and poly(DEE_0.8_‐MMA) (based on CA); (d) Transmittance of FTO/PB/poly(DEE_0.8_‐MMA)/FTO ECD at different voltages with the digital photos in coloration (2 V) and bleaching (−3.5 V) states; (e) Transmittance at 633 nm of ECD over 2000 cycles (voltage range of −3.5 to 2.0 V).

To evaluate the potential practical application of poly(DEE‐MMA) in ECDs, semi‐electrochromic devices with the configuration of PB||poly(DEE_0.8_‐MMA)||FTO were fabricated and compared with devices based on poly(DEE_0_‐MMA). The bleaching and coloration times were defined as the durations required to reach 90% of the peak or valley transmittance, respectively. The transmittance‐vs.‐time curves for two samples, as shown in Figure 5c, show that the coloring/bleaching time of the device using poly(DEE_0.8_‐MMA) decreases from 121.7 s/43.5 s to 14.7 s/21.1 s, respectively. Transmittance of the devices at various applied voltages is shown in Figure 5d, accompanied by digital photographs of the semi‐device. The maximum optical modulation amplitude (ΔT) is approximately 54.8% at 633 nm under a voltage range of −3.5 to 2 V. A representative switching action of the ECD is shown in Video S1. Furthermore, the calculated coloring efficiency (CE) of the semi‐ECD is 62.99 cm^2^ C^−1^ (Figure S22), which is considered acceptable for a PB layer (87.8 cm^2^ C^−1^ in PC‐LiClO_4_) [59].

To further investigate diffusion mechanisms, cyclic voltammetry (CV) was performed on the ECD at various scan rates to determine the effective diffusion coefficient (*D*_Li+_) using the Randles‐Sevcik equation. The two redox couples and one reduction peak were revealed (Figures S23 and S24). The *D*_Li+_ values for bleaching and coloration range from 5.87 × 10^−11^ to 1.52 × 10^−9^ cm^2^ s^−1^ (Table S5), exceeding those reported in previous studies [60, 61]. The ECD using poly(DEE_0.8_‐MMA) as the electrolyte demonstrated excellent reversibility and stability, thereby contributing to the long‐term cycling durability of devices. The cyclic stability of the ECD was assessed by in situ monitoring of transmittance at 633 nm during a chronoamperometry (CA) test under bias voltages of −3.0 to 1.5 and −3.5 to 2.0 V, as shown in Figure 5e and Figure S25. Although a gradual decrease in the transmittance of the bleaching state was observed with increasing cycle number, under a voltage range of −3.0 to 1.5 V, an initial transmittance (T%) of 48.1% is observed, and 40.4% is retained after 3000 cycles, corresponding to a retention of 84%. Under a wider voltage window of −3.5 to 2.0 V, an initial T% of 53.5% is measured, and 43.6% is maintained after 2000 cycles, corresponding to a retention of ∼81%, indicating stable switching performance. Meanwhile, the cyclic stability of the ECD at 60°C was further investigated by monitoring the current intensity (Figure S26) [62]. The ECD exhibits exceptional stability over a duration of 4000 stability cycles, thereby signifying that the deep eutectic polymer demonstrates remarkable high‐temperature durability when employed in conjunction with lithium perchlorate. Additionally, the ECD assembled here exhibits superior electrochromic performance compared to that reported in previous literature (Table S6), demonstrating that the synthesized poly(DEE_0.8_‐MMA) possesses favorable electrochemical and optical properties, indicating its potential as a promising alternative to liquid electrolytes in ECD applications.

## 3 Conclusion

In conclusion, a novel polymerizable PDEE strategy is presented based on the in situ copolymerization of a ternary DEE and MMA. The obtained poly(DEE‐MMA) electrolyte exhibits an ionic conductivity of 1.43 mS cm^−1^, a wide electrochemical window of 4.6 V, and a high t_Li+_ of 0.83. It is demonstrated that strong coordination interactions and hydrogen bonding between the hydrogen‐bonding donor (AM), and the hydrogen‐bonding acceptors (LiClO_4_ and SN) facilitate the dissociation of lithium ions from anions, thereby accelerating Li^+^ transport. The polymer backbone, formed by the dual‐skeleton effect of AM and MMA, endows the solid polymer electrolyte with unique optical characteristics and outstanding mechanical properties, including a tensile strength of 17.8 MPa. These enhanced properties allow the Li||LiFePO_4_ battery to achieve 120 cycles with 83% capacity retention, while the electrochromic device maintains 2000 coloration‐bleaching cycles, retaining 80% of its initial optical modulation amplitude, demonstrating excellent long‐term stability. This work provides a novel strategy for synthesizing solid‐phase deep eutectic electrolytes and offers guidance for the design of high‐performance electrolytes for advanced multifunctional electrochemical devices.

## Conflicts of Interest

The authors declare no conflict of interest.

## Supporting information




**Supporting File 1**: advs76363‐sup‐0001‐SuppMat.pdf.

**Supporting File 2**: advs76363‐sup‐0002‐VideoS1.mp4.

## Data Availability

The data that support the findings of this study are available on request from the corresponding author. The data are not publicly available due to privacy or ethical restrictions.
