# Supplementary material for: High‐Performance Transparent Solid Polymer Electrolyte Based on Copolymer of Deep Eutectic Electrolyte and Methyl Methacrylate for Electrochemical Devices
Source: Adv Sci (Weinh). 2026 Jun 28;13(53):e76363. doi: 10.1002/advs.76363 (PMC13337015; doi:10.1002/advs.76363)
Supplement: Supplementary file 1 [file ADVS-13-e76363-s002.pdf]

Supporting Information

High-Performance Transparent Solid Polymer Electrolyte Based on Copolymer of Deep Eutectic Electrolyte and methyl methacrylate for Electrochemical Devices

Tingting Chen[a], Sijia Han[a], Likun Wang[b], Sainan Ma[b], Liping Zhang[a], Yong Liu[a]*, Gaorong Han[a, b]*

[a] *School of Materials Science and Engineering, Zhejiang University, Hangzhou, 310058, China*

[b] *Ningbo Global Innovation Center, Zhejiang University, Ningbo, China, 315100*

Materials

Methyl methacrylate (MMA, 99.5%, Aladdin), acrylamide (AM, 98.0%, Sinopharm Chemical Reagent Co., Ltd), succinonitrile (SN, 99%, Sigma), and lithium perchlorate ($LiClO_4$, 99.9%, Aladdin), 2'-azobis(2methylpropionitrile) (AIBN, 99%, Aladdin), triallylisocyanurate (TAIC, 95%, Maya), were used as purchased without further purification. N-methyl-2-pyrrolidone (NMP, 99%, Aladdin), polyvinylidene fluoride (PVDF, Aladdin), Super P (XC-72R, Rhawn), $LiFePO_4$ (LFP, 98%, Shanghai Yuanye Bio-Technology Co., Ltd.), glass fiber (GF/A, Whatman), and carbon-coated aluminum foil were purchased from Badoglio corporation. Lithium metal (thickness 0.45 mm × diameter 15.6 mm) was purchased from China Energy Lithium Co., Ltd.

Preparation of poly(DEE-MMA) electrolytes

Polymerizable ternary eutectic mixtures were prepared by mixing AM, SN, and $LiClO_4$ with varying molar ratios. Initially, AM and SN were mixed and stirred at molar ratios of 1:x (x = 2, 3, 4, 8). Subsequently, $LiClO_4$ was added to achieve the particular compositions of AM: SN: $LiClO_4$ = 1: x: y (y=0, 0.2, 0.4, 0.6, 0.8, 1.2). The mixtures, designated as AS_xL_y, were stirred at 60 °C for 4 h until the transparent ternary eutectic mixtures were obtained.

DEE-MMA monomers were prepared by mixing $AS_4L_{0.6}$ and MMA in volume ratios of 0:1, 0.2:0.8, 0.4:0.6, 0.6:0.4, 0.8:0.2, and 1:0, respectively. The mixtures were stirred at room temperature until the transparent precursor solutions, designated as DEE_Z-MMA monomer (where Z = 0, 0.2, 0.4, 0.6, 0.8, and 1, respectively, and which denotes the volume fraction of DEE), were obtained. Specifically, DEE_0-MMA was prepared as a DEE-free control by mixing 2 ml of SN with 0.22 g of $LiClO_4$ with 1ml of MMA monomer, without the inclusion of DEE. Subsequently, 1 wt% AIBN as the initiator and 2 wt% of TAIC were dissolved in the DEE_Z-MMA monomers at room temperature. In order to achieve complete polymerization, the mixtures were heated at 60 °C for 6 h,

designated as poly(DEE_Z-MMA) (where Z = 0, 0.2, 0.4, 0.6, 0.8, and 1, respectively).

Preparation of the lithium metal battery

For coin-cell battery testing, the cathodes were prepared using the traditional slurry-coating method. LFP, Super P, and PVDF were combined in a weight ratio of 80:10:10. A few drops of NMP solvent were added to create a uniform slurry. The slurry was then applied to a carbon-coated aluminum foil and vacuum-dried at 70 °C for 12 hours. The dried cathodes were punched into 14 mm diameter discs with an active material loading of approximately 2.5 mg/cm^2. Lithium metal served as the anode, and GF/A was used as the separator. The GF/A separator was employed specifically to facilitate the in-situ polymerization process and ensure consistency in cell assembly[1]. All coin-cell batteries for electrochemical testing were assembled by injecting 80 μL of the respective electrolyte precursor during the process. After adding the precursor, the batteries were heated at 60 °C for 6 hours to complete polymerization. The commercial electrolyte 1 M $LiClO_4$ was in a solvent of propylene carbonate (PC), as the positive controls. All procedures were conducted in a glovebox with an argon atmosphere (H_2O, O_2 $\leqslant$ 1 ppm).

Preparation of the electrochromic device

The electrochromic electrode was prepared by depositing Prussian blue (PB) films on FTO glass using the chemical bath deposition method, as previously reported[2]. A separate piece of FTO glass was employed as the counter electrode of the electrochromic device (ECD). The ECD was assembled by sandwiching the poly(DEE-MMA) between the electrochromic and counter electrodes. Subsequently, the assembly was heat-treated at 60 °C for 6 hours to complete polymerization. The free edges of the substrates were then secured with the conductive copper tape to ensure reliable electrical contact.

Material characterizations

Differential scanning calorimetry (DSC) analysis of the AS_xL_y ternary system was performed from -50 °C to 40 °C at a heating rate of 10 °C/min using a DSC 2500 instrument (TA Instruments, USA). The viscosity of the AS_xL_y ternary DEE was measured in sweep mode over a frequency range of 0.1 ~ 10 Hz at 25 °C with a MARS 60 rotational rheometer (HAAKE, Germany). ^{1}H NMR and ^{13}C NMR spectra were obtained by using a Bruker spectrometer operating at 600 MHz. The intra- and inter-molecular interactions were analyzed using Fourier Transform Infrared spectroscopy (FTIR, Nicolet iS50, Thermo Fisher) in the range of 400 cm^{-1} ~ 4000 cm^{-1}. X-ray diffraction (XRD, LabX XRD-6000, SHIMADZU) data were collected at room temperature over the range of $5° \leq 2\theta \leq 80°$ at a scanning rate of 4°/min and a step length of 0.02° with Cu Kα radiation. Scanning electron microscopy (SEM, Gemini 360) was used to characterize the morphology of the samples with an accelerating voltage of 5.00 kV and magnifications of ×1000 and ×20k. Using ASTM International standard D1002 as a reference, the lap-shear strength of poly(DEE-MMA) electrolyte-bonded glasses with a 13 mm × 25 mm area was measured using a universal material testing machine (Zwick/Roell Z020, Germany). The tensile strength of the poly(DEE-MMA) was also measured using the same universal material testing machine at a test speed of 50 mm/min and 25 °C. The optical spectra of poly(DEE-MMA) and ECDs were measured over 200 nm to 1000 nm using a UV-Vis spectrophotometer (UV-Vis spectroscopy, Cary 5000, Agilent).

Electrochemical Measurements

The electrochemical impedance spectroscopy (EIS), lithium-ion transference number (t_{Li^+}), cyclic voltammetry (CV), and linear sweep voltammetry (LSV) were performed on a CHI660 electrochemical workstation (Chenhua, Shanghai). The ionic conductivity of the poly(DEE-MMA) electrolyte was measured by impedance spectroscopy over a

frequency range from 0.1 Hz to 10^6 Hz in stainless steel cells, with temperatures ranging from 25 °C to 100 °C. The t_{Li+} was measured and calculated by a direct current (DC) voltage of 10 mV and alternating current (AC) impedance measurement using Li||Li cells at room temperature, using the following equation:

$$t_{Li^+}=\frac{I_{ss}(\Delta V\text{-}I_0R_i^0)}{I_0(\Delta V\text{-}I_{ss}R_i^{ss})} \quad (1)$$

where I_0 is the initial current, I_{ss} represents the steady-state current, and R_i^0 and R_i^{ss} are the initial charge-transfer resistances and steady charge-transfer resistances, respectively. ΔV is the applied polarization voltage.

The electrochemical stability window of the electrolytes was measured by the LSV method in SS||SE cells within a voltage range of 0 V ~ 6.0 V at a scan rate of 10 mV/s. Galvanostatic discharge-charge tests of coin cells were conducted using a Neware battery measurement system (Neware, Shenzhen, China). Li was plated and stripped in Li|Li symmetric cells at room temperature with a constant current density of 0.1 mA/cm^2. The current densities for Li||LFP cells were set to 1C = 170 mA/g. Tests were conducted on the LFP cathode over a voltage range of 2.5 V to 4.2 V at various specific currents.

DFT calculations

The calculations were performed using Gaussian 16 software with the B3LYP/6-31+g(d) method. To determine the optimal geometry for each compound, geometry optimization calculations were performed using the Def2-SVP basis set. Additionally, single-point energy calculations were performed using a larger basis set, Def2-TZVP. Then, the interaction energy between $LiClO_4$, SN, and AM was calculated by the following equation:

$$\Delta E=E_{complex}\text{-}(E_{AM}+E_{LiClO_4}+E_{SN}) \quad (2)$$

The structures of molecules, including PAM, PMMA, and PMMA-AM, were optimized at the B3LYP/6-31+g(d) level of theory. These structures were characterized as local energy minima on the potential energy surface. In the simulation, we simplified PMMA

and PAM into model compounds that represent their structural units. The only difference between them and the original polymers is the degree of polymerization. We assume that the establishment of model compounds follows the same rules as the original polymers.

The electrostatic potential (ESP) calculations were performed with the ORCA program package (version 6.0)[3]. The hybrid B3LYP density functional was utilized for geometry optimizations. The all-electron triple-quality Def2-TZVP basis sets were applied for all elements. All the geometries were fully optimized without symmetry constraints. ESP of AM, SN, and $LiClO_4$ molecules were generated by Multiwfn[4]. The electrostatic potential distributions are visualized using VMD.

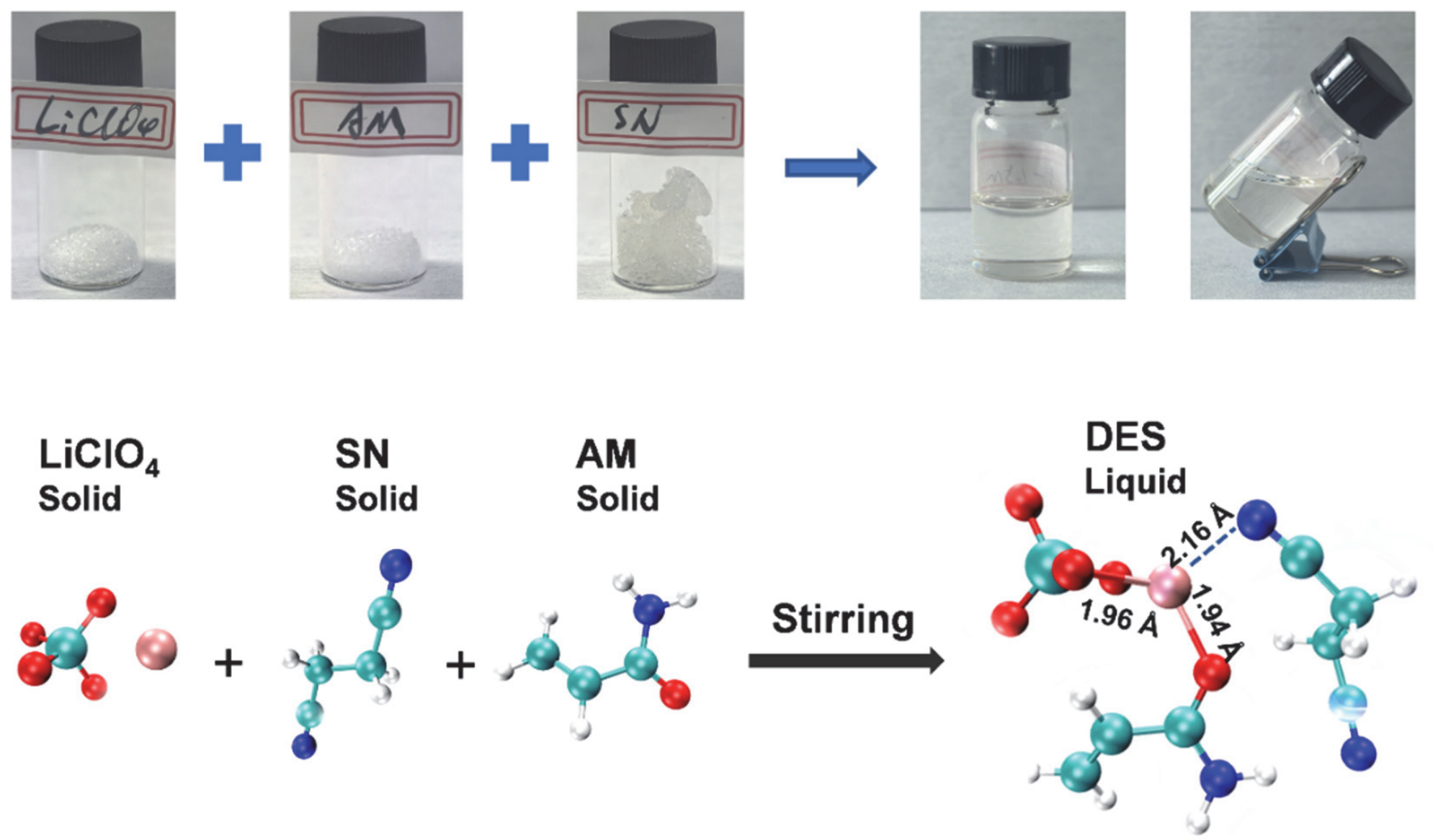

Figure S1. (a) Optical images of DEE through the mixing of three components; (b) Structure diagram and inter-molecular distances of DEE through DFT calculations.

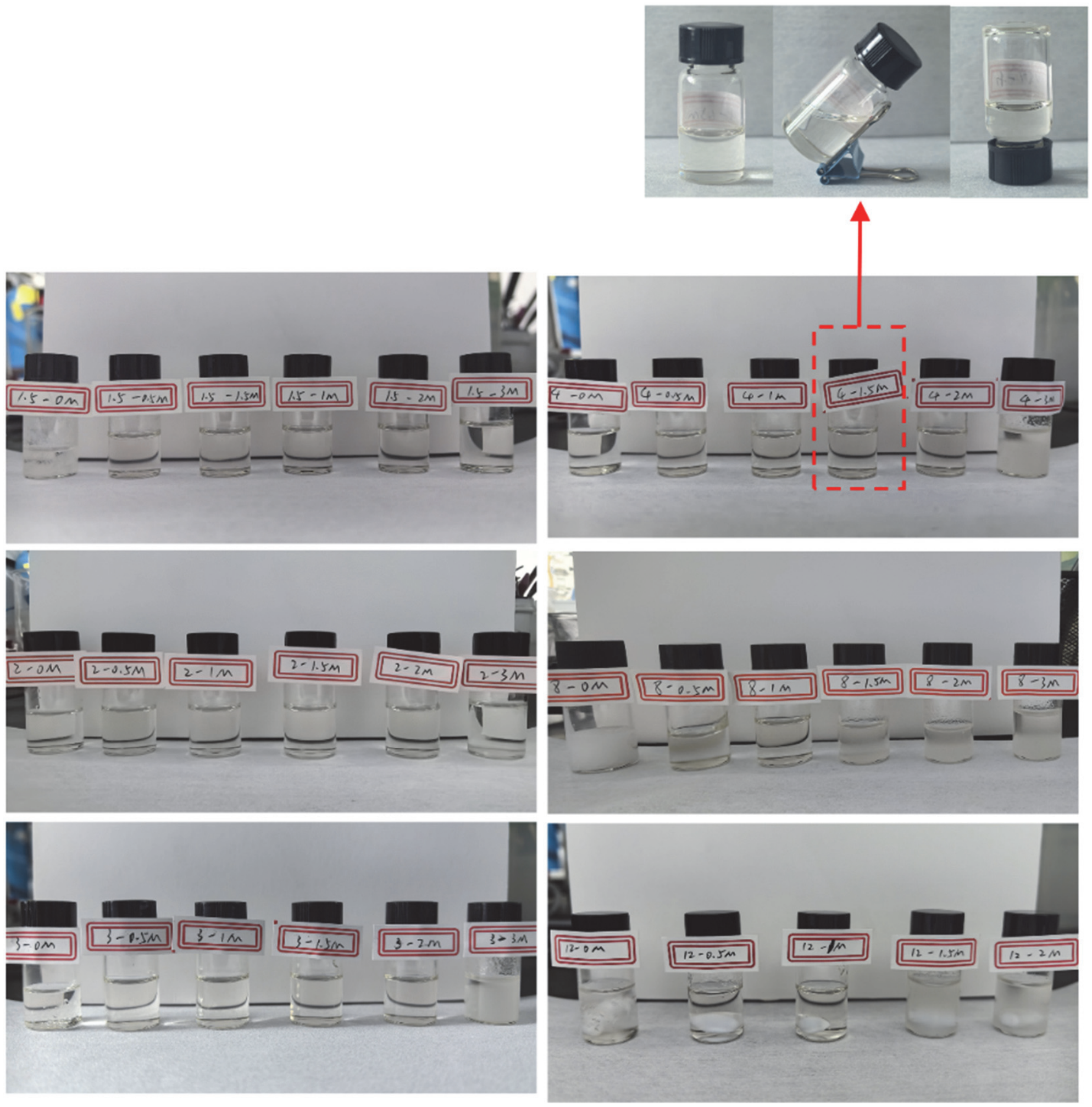

Figure S2. A portion of the digital photographs of the DEEs prepared with various ternary component ratios at room temperature.

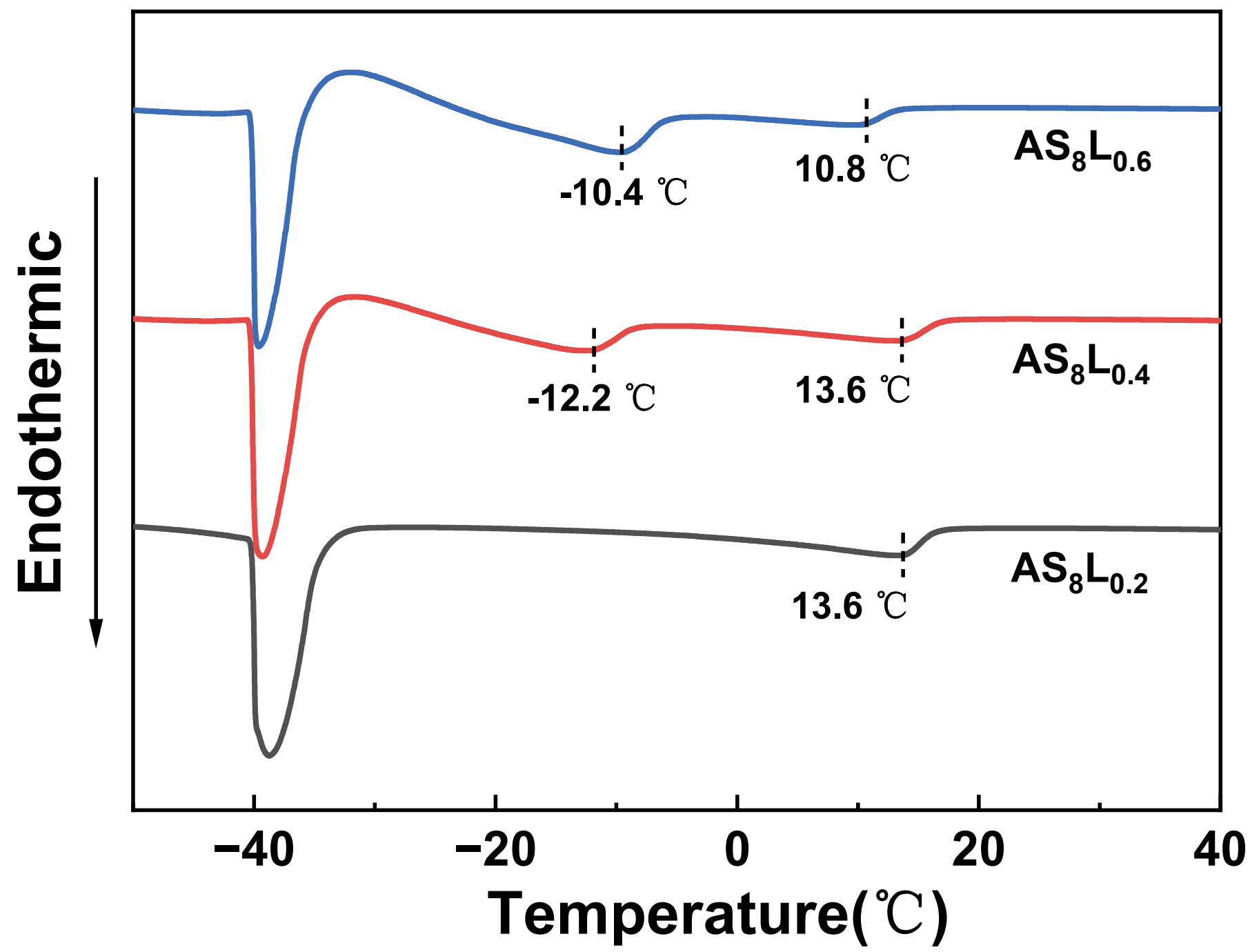

Figure S3. DSC spectra of AS_8L_y (T_m: melting temperature).

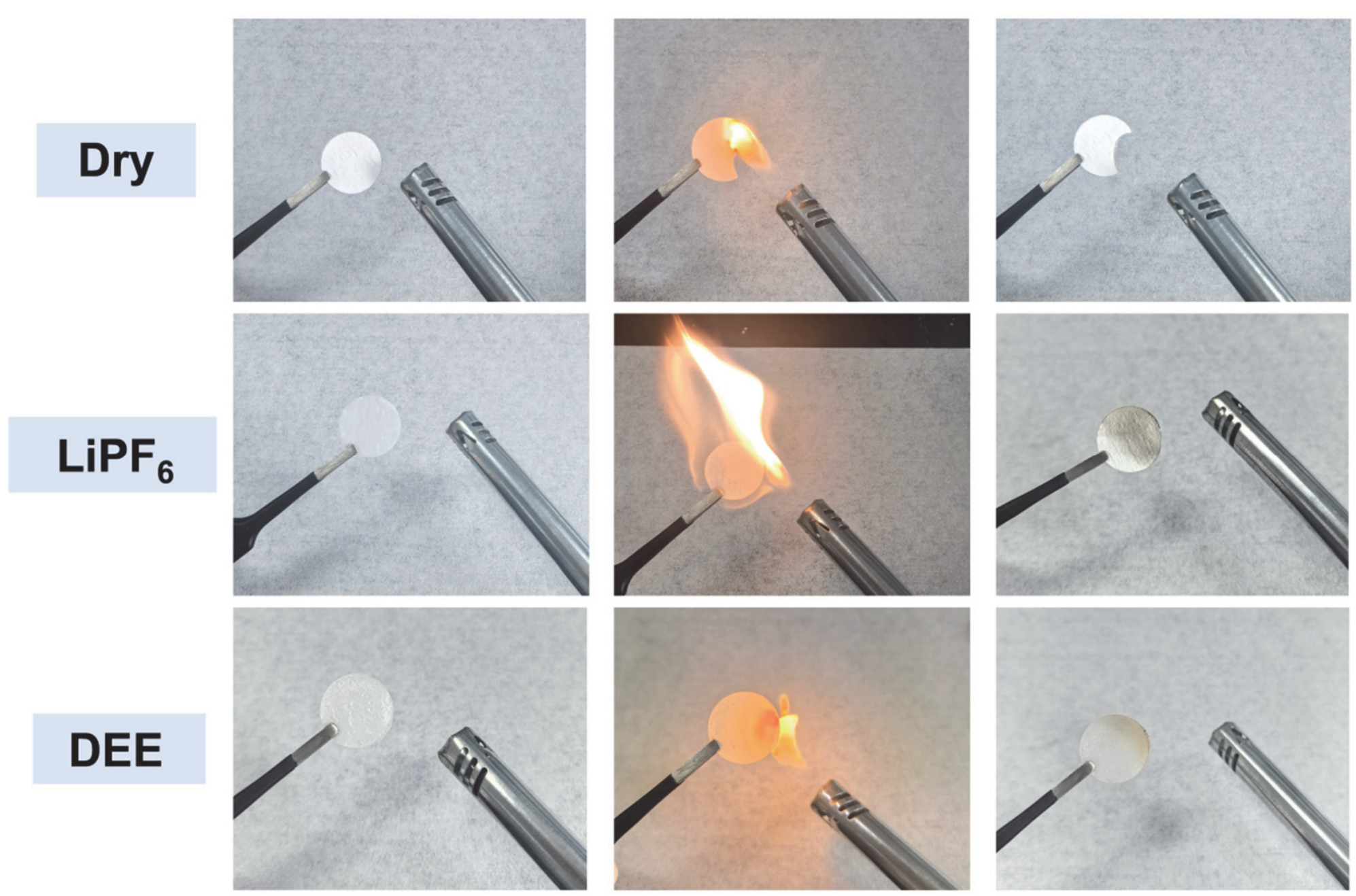

Figure S4. Flammability tests of glass fiber separators soaked in different electrolytes.

Table S1. ^{1}H NMR data of AM, SN, and two types of ASL

Type	Chemical shift/ppm			
	AM	SN	$AS_4L_{0.6}$	$AS_4L_{0.8}$
a	5.65	-	5.74	5.74
b	6.24	-	6.24	6.24
c	6.31	-	6.56	6.54
d	7.22	-	7.06	7.09
e	-	2.92	2.83	2.83

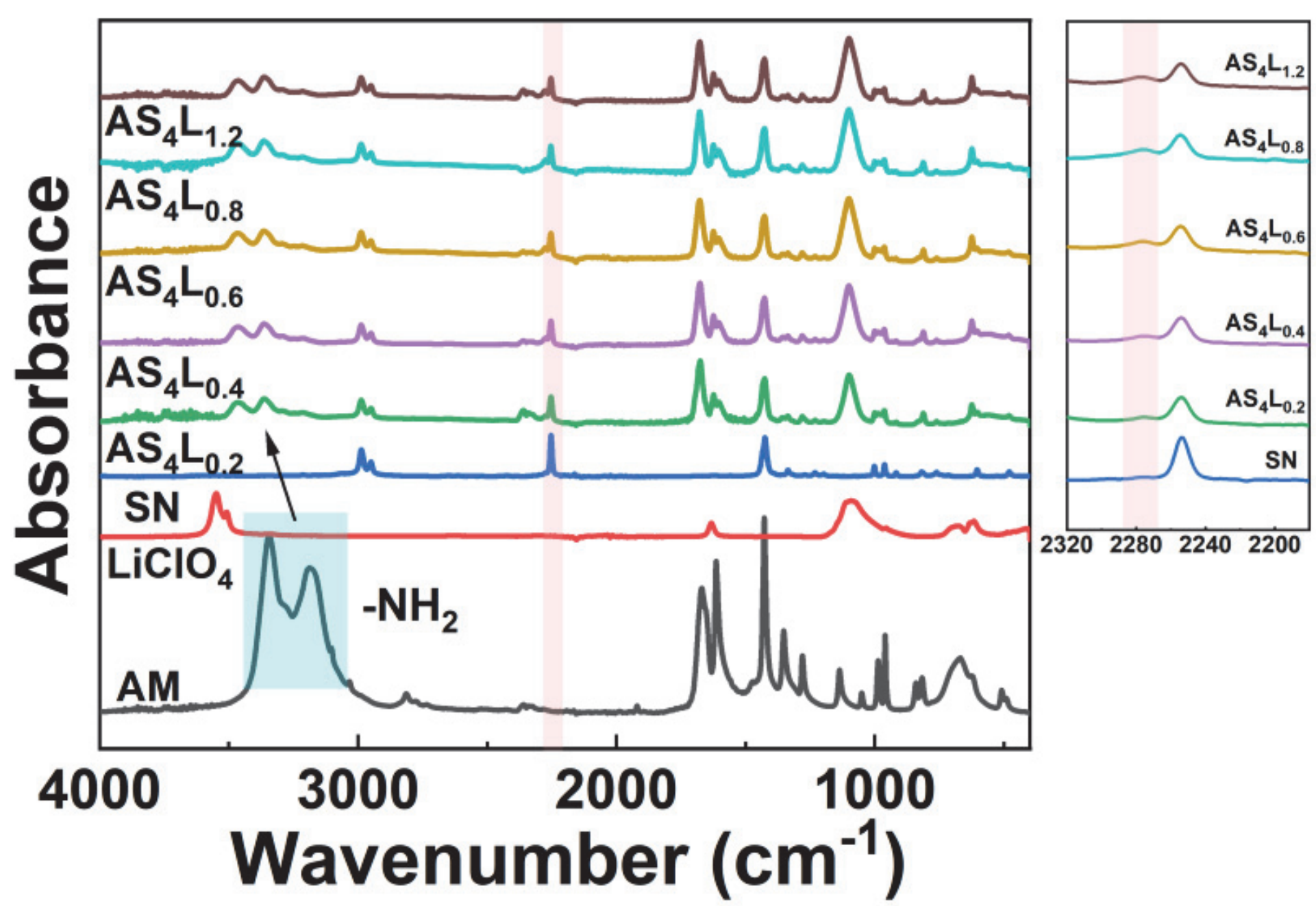

Figure S5. FTIR spectra of the $LiClO_4$, AM, and SN of AS_4L_y samples and corresponding to the nitrile group of AS_4L_y samples.

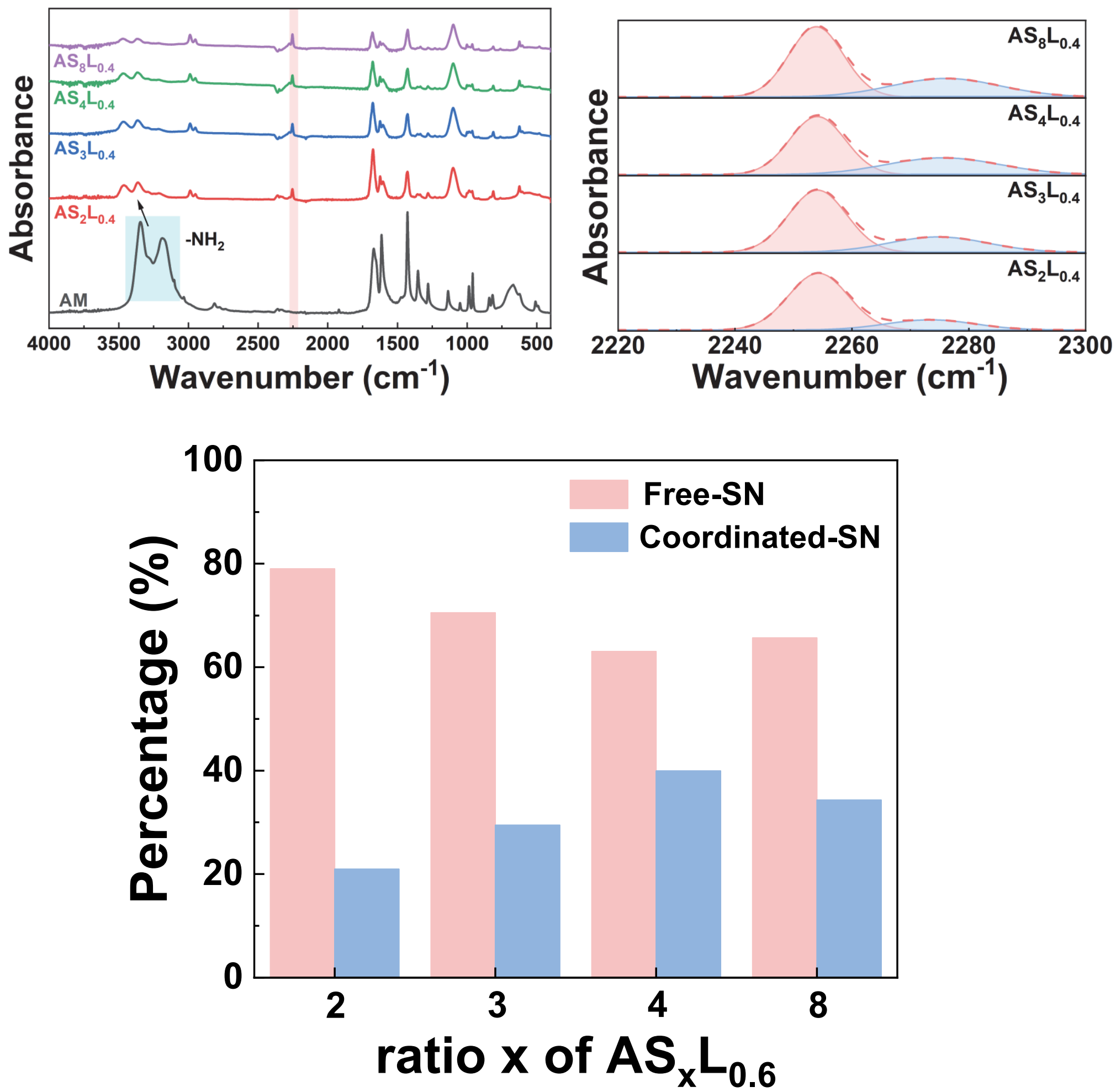

Figure S6. (a) FTIR spectra of cyanide peaks in $AS_xL_{0.6}$ samples with different SN ratios; (b) SN species (Free-SN and Coordinated-SN) distributions in $AS_xL_{0.6}$ samples with different SN ratios obtained from the fitted FTIR spectra.

Note 1: The integrated area of the free SN peak at 2254 cm^{-1} decreases and then increases with increasing SN content in $AS_xL_{0.6}$. The initial decrease in peak area indicates a reduction in free-SN molecules, due to strong interactions between Li^+ and the C≡N group in SN, which promotes lithium salt dissociation and enhances ionic conductivity. These findings are supported by the measured ionic conductivity, with AS_4L_y exhibiting the highest values (Figure S7).

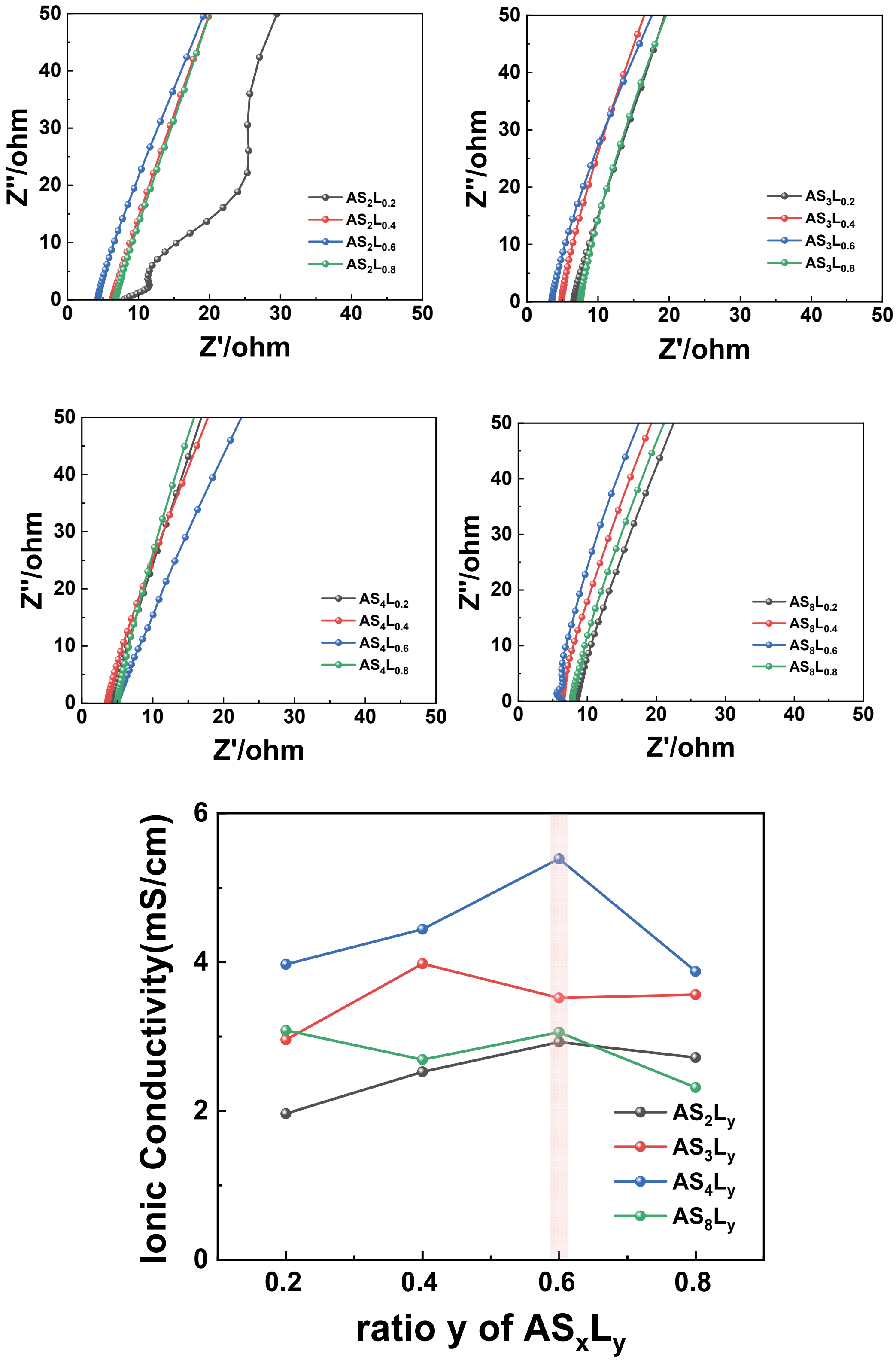

Figure S7. Electrochemical impedance spectra (EIS) of AS_xL_y at 25 °C and the yielded ionic conductivities.

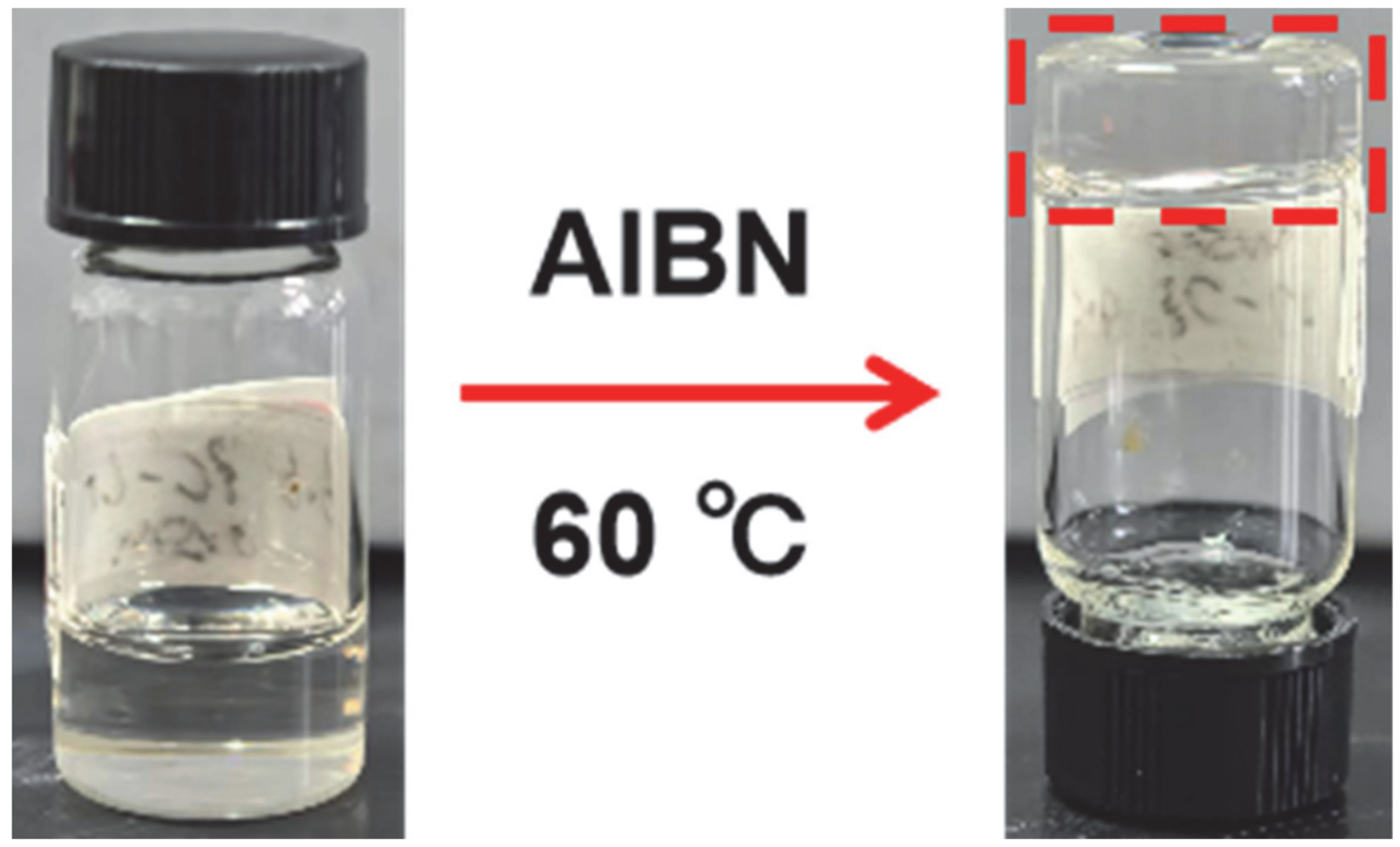

Figure S8. Optical images of DEE-MMA monomer and poly(DEE-MMA).

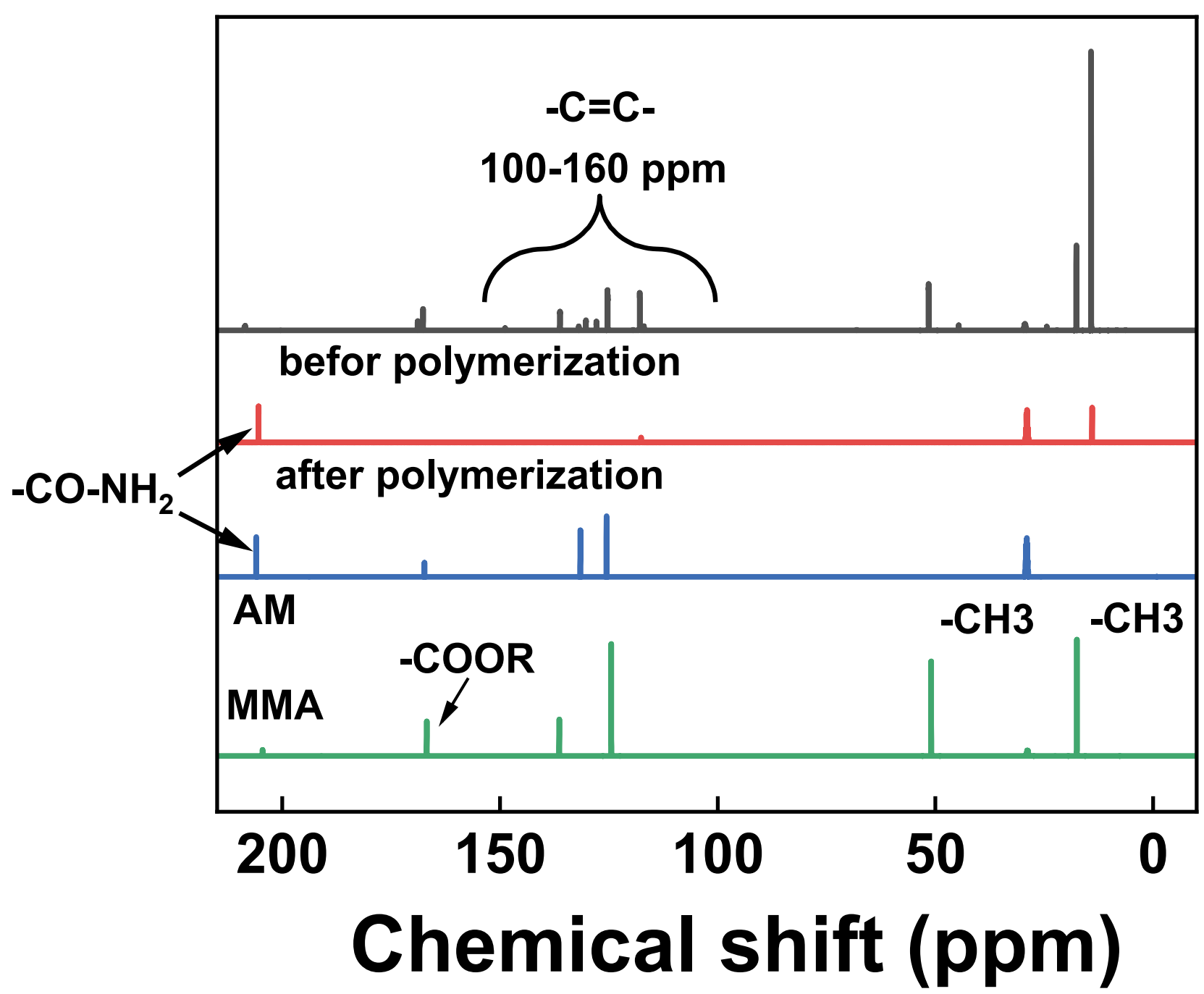

Figure S9. ^{13}C NMR spectra of poly(DEE-MMA) electrolyte before and after polymerization in Acetone-d6.

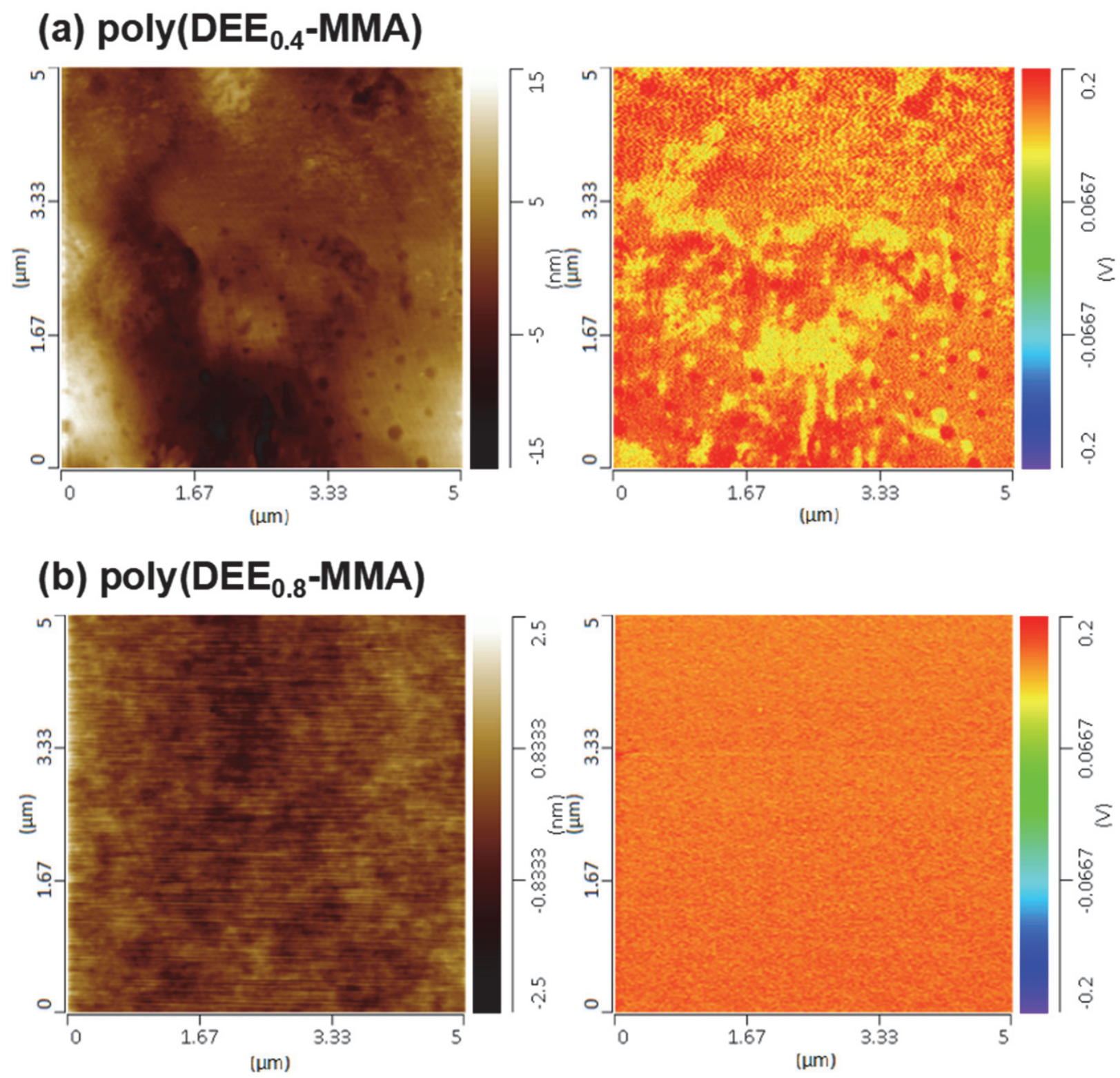

Figure S10. AFM-IR images of poly($DEE_{0.4}$-MMA) and poly($DEE_{0.8}$-MMA) samples at 2254 cm^{-1}.

Note 2: The distribution of the DEE-rich phase was characterized using an atomic force microscopy-infrared (AFM-IR) imaging at a wavenumber of 2254 cm^{-1}, corresponding to the C≡N stretching vibration of SN. For poly($DEE_{0.4}$-MMA), the presence of interconnected C≡N domains confirms the formation of continuous ion transport pathways. Even at relatively low DEE concentrations, molecular aggregation is initiated, leading to the formation of continuous transport pathways, consistent with the observed sharp increase in ionic conductivity. In contrast, samples with higher DEE content (0.8) display more uniformly and densely distributed C≡N signals, indicating that the transport network has reached a saturated state within the polymer matrix. Consequently, further addition of DEE contributes minimally to the formation of new conduction pathways, thereby resulting in the observed inflection point in conductivity and its subsequent gradual increase.

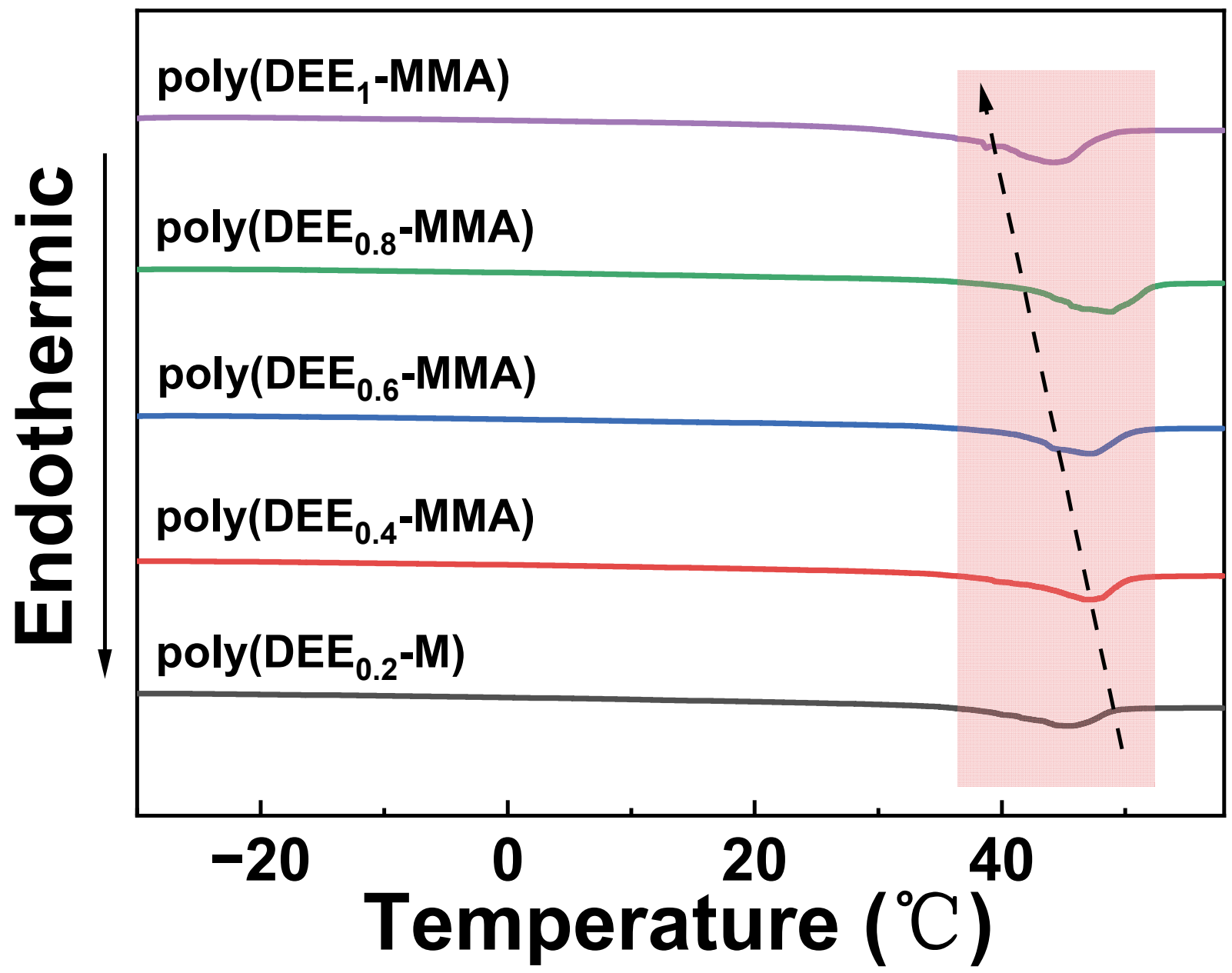

Figure S11. DSC thermal profiles of poly(DEE-Z-MMA) from -30 to 50 ºC, showing T_g.

Note 3: poly(DEE$_Z$-MMA) samples exhibit a change in slope at the conductivity transition temperature (close to Tm observed in the DSC as shown in Figure S10).

Ionic conductivities (σ_{dc}) of poly(DEE$_Z$-MMA) below the glass transition temperature (T_g) were fitted using the Vogel-Tamman-Fulcher (VTF) equation:

$$\sigma_{DC}=\frac{A}{T^{1/2}}\exp\left[-\frac{B}{(T-T_0)}\right] \qquad \text{Equation S1}$$

where A is a pre-exponential factor related to the concentration of charge carriers, B represents the apparent activation energy, R is the ideal gas constant, T is the absolute temperature, and T_0 is the Vogel temperature associated with T_g.

Ionic conductivities (σ_{dc}) of poly(DEE$_Z$-MMA) above the glass transition temperature (T_g) were fitted using the Arrhenius equation:

$$\sigma=\sigma_0\exp\left(-\frac{E_a}{kT}\right) \qquad \text{Equation S2}$$

where σ_o is the pre-exponential factor, E_a is the activation energy, k is the Boltzmann constant, and T is the temperature.

The parameter information obtained from fitting each sample is shown in the table below.

Table S2. Low-temperature VFT fitting parameters (D, B, T_0) and high-temperature Arrhenius fitting parameter (E_a)

Sample	D	B (K)	T_0 (K)	Ea (eV)
poly(DEE_0-MMA)	0.42	115.5	276	0.26
poly($DEE_{0.2}$-MMA)	0.37	102.6	273	0.24
poly($DEE_{0.4}$-MMA)	0.31	83.7	271	0.20
poly($DEE_{0.6}$-MMA)	0.28	77.2	275	0.18
poly($DEE_{0.8}$-MMA)	0.08	23.1	284	0.11
poly(DEE_1-MMA)	0.29	78.7	273	0.16

Note 4: In the high-temperature region, where polymer chains undergo segmental relaxation, the conductivity is described by Arrhenius behavior. The calculated activation energy (E_a) represents the energy barrier for ion migration along fast-conduction pathways facilitated by internal small molecules and polymer backbone functional groups. Ea is observed to decrease systematically with increasing DEE content, with poly($DEE_{0.8}$-MMA) exhibiting the lowest energy barrier of 0.09 eV, which facilitates rapid ion transport. Conversely, in the low-temperature region, the non-linear behavior is well described by the Vogel-Tammann-Fulcher (VTF) model. The extracted strength parameter ($D = B/T_0$) serves as a qualitative measure of the system's deviation from Arrhenius behavior[5,6]; the poly($DEE_{0.8}$-MMA) electrolyte exhibits a minimum D value of 0.08, indicating a reduced dependence on coordinated segmental motion and a higher contribution from molecular-level transport even at lower temperatures, contributing to the enhanced overall ionic conductivity observed for the poly($DEE_{0.8}$-MMA) system.

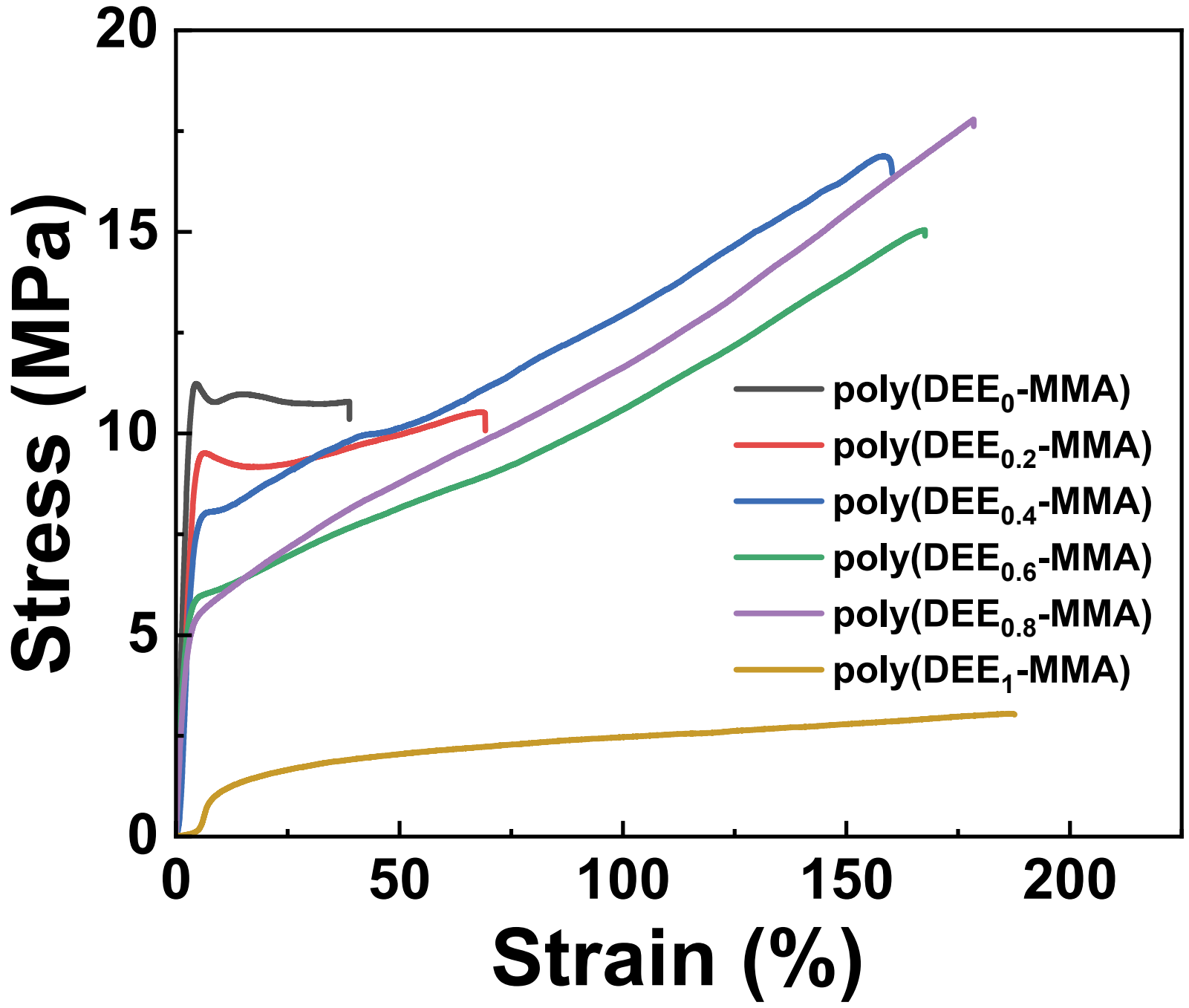

Figure S12. Stress-strain curves for different electrolyte samples tested at a speed rate of 20 mm min^{-1}.

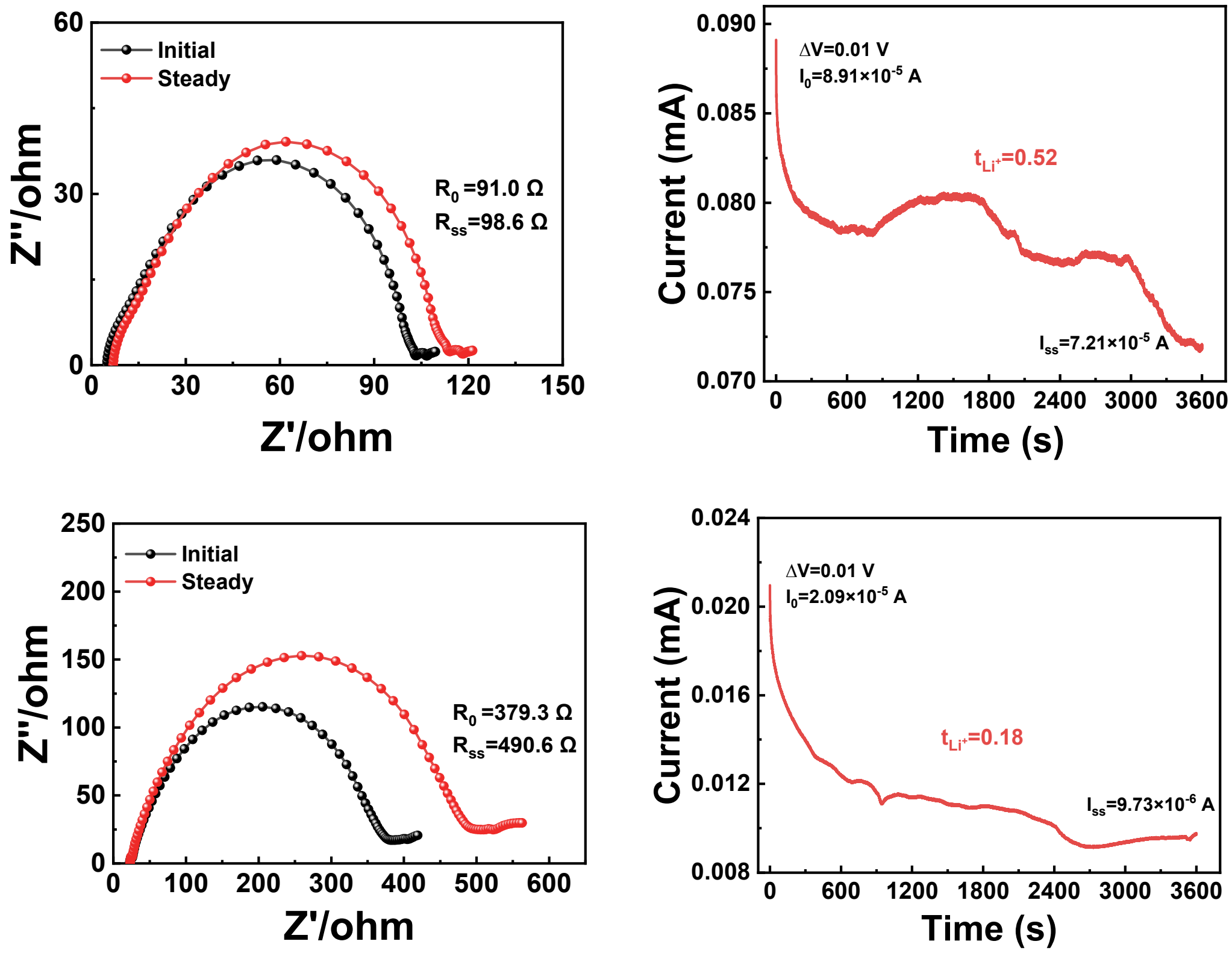

Figure S13. (a) and (b) Chronoamperometry profile of Li|1 M $LiClO_4$ in PC |Li cells under a 10 mV polarization voltage with EIS spectra before and after polarization. (c) and (d) Chronoamperometry profile of Li|poly(DEE_0-MMA)|Li cells under a 10 mV polarization voltage with EIS spectra before and after polarization.

Note 5: To contextualize the ion transport behavior of the developed electrolytes, the t_{Li+} of two reference systems were measured for comparison (Figure S13). The conventional liquid electrolyte (1 M $LiClO_4$ in PC) exhibits a t_{Li+} of approximately 0.5, characteristic of dual-ion conducting systems where the absence of selective ion–matrix interactions leads to comparable mobilities of cations and anions. In contrast, the pristine PMMA-based electrolyte without deep eutectic components P(DEE_0-MMA) shows a significantly lower t_{Li+} of ~ 0.2, attributed to the strong coordination between Li^+ and carbonyl (C=O) groups, which imposes persistent hopping barriers and restricts long-range cation migration despite promoting salt dissociation.

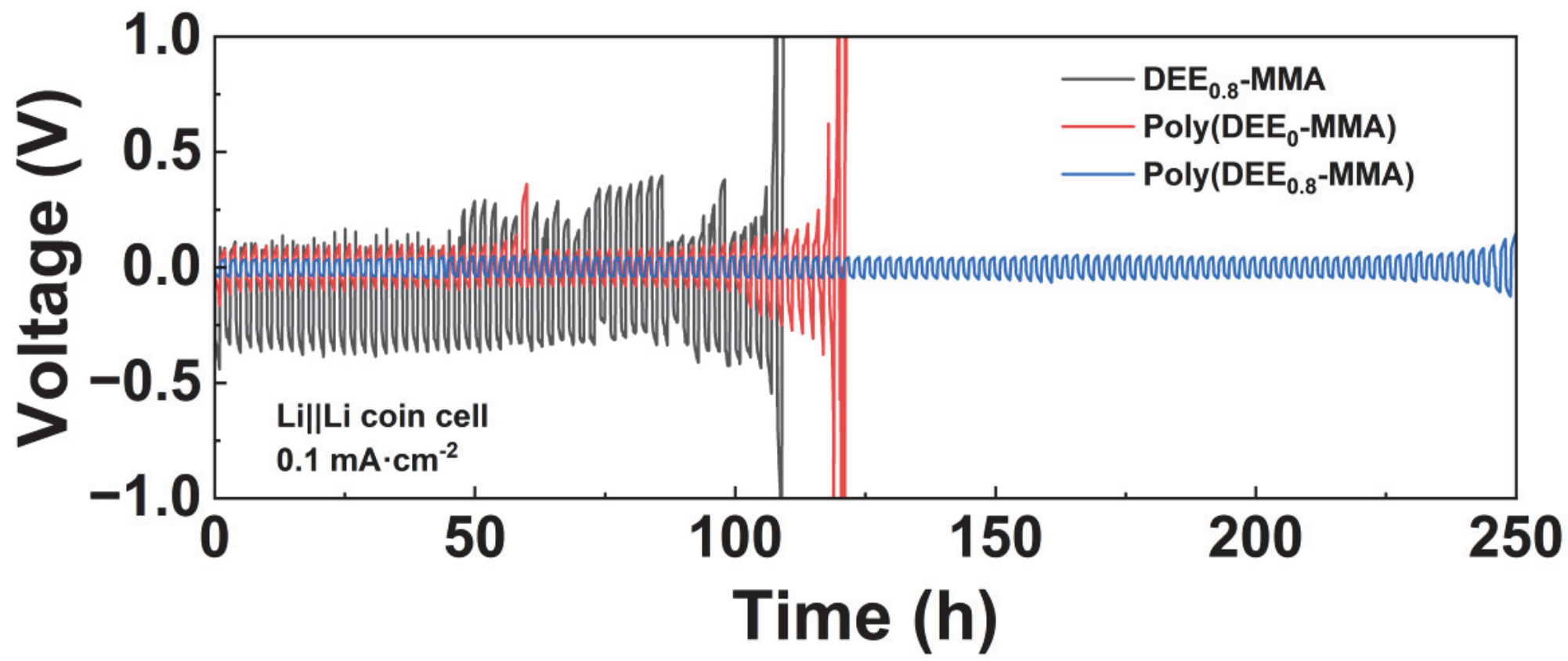

Figure S14. Galvanostatic cycling curve of a Li symmetric cell at a current density of 0.1 mA cm^{-2}

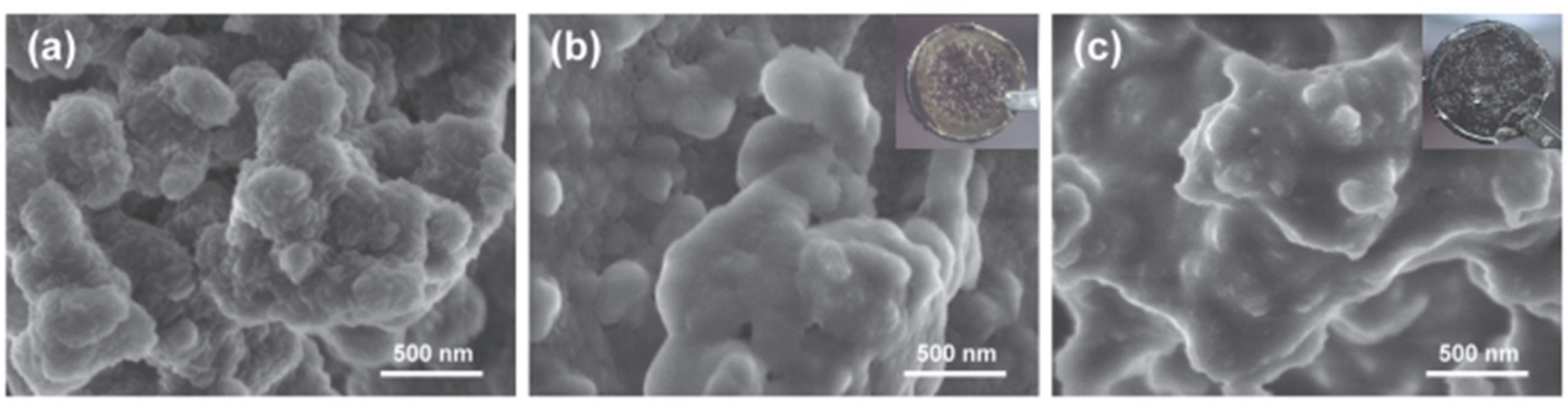

Figure S15. SEM images of lithium metal anode in the (a) bare Li; (b) poly($DEE_{0.8}$-MMA) and (c) $DEE_{0.8}$-MMA after plating/stripping cycles. Inserts show the photos of lithium metal anode after 250 h and 120 h.

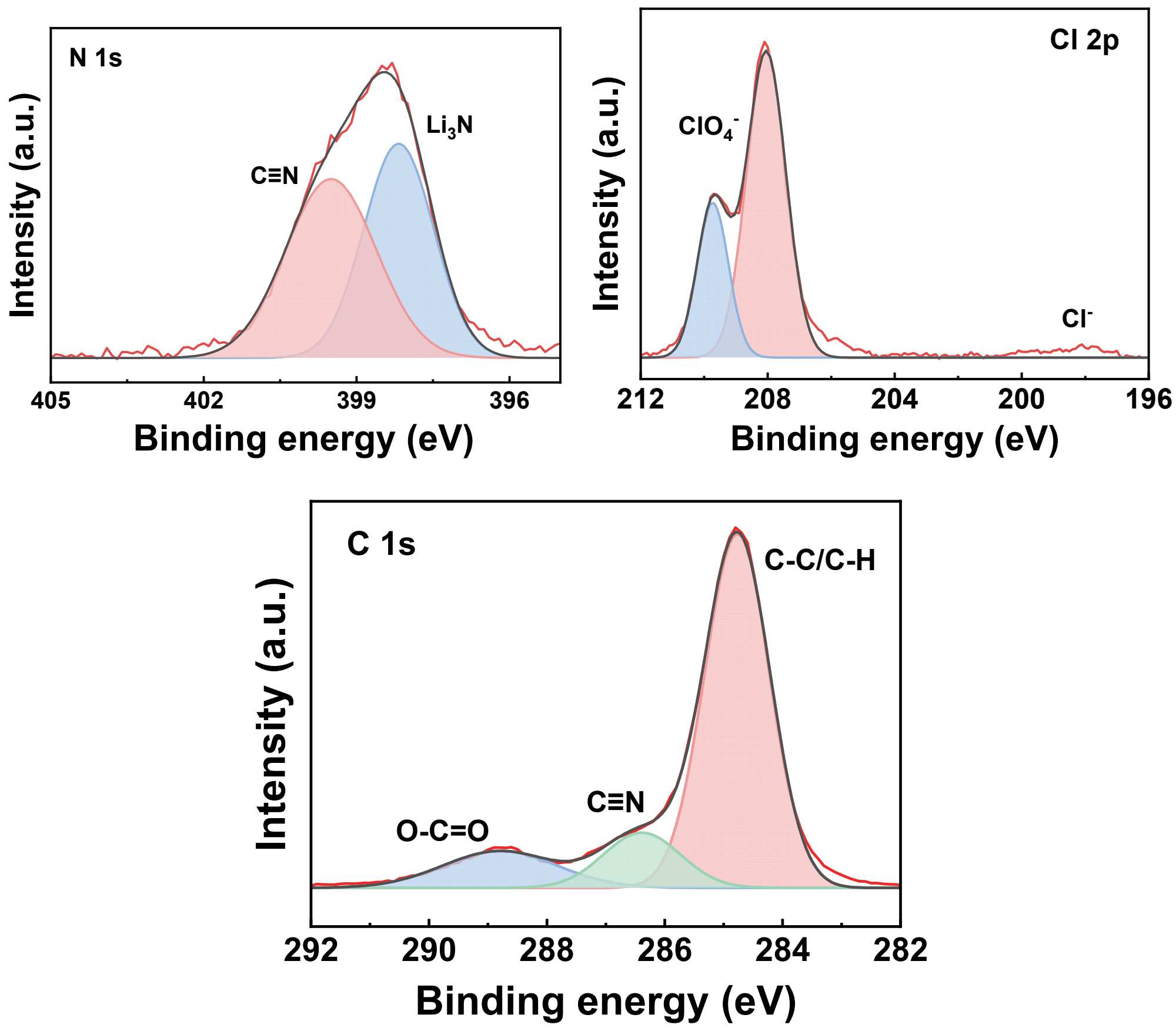

Figure S16. (a-c) XPS analysis (N 1s, Cl 2p, and C 1s spectra) of Li metal anode in poly($DEE_{0.8}$-MMA) after plating/stripping cycles.

Note 6: Through scanning electron microscopy (SEM), it was found that the use of poly($DEE_{0.8}$-MMA) maintained a smooth and dense lithium metal surface, forming a thin protective layer of Li_3N that preserved the original fine-particle morphology. The continuous reaction between the Li metal and the non-polymerized $DEE_{0.8}$-MMA electrolyte caused a thicker organic layer on the lithium surface, obscuring the original microstructure. These results showed that the poly(DEE-MMA) electrolyte enabled a uniform deposition of Li on the anode, effectively inhibiting side reactions and the growth of Li dendrites.

In addition to the partial decomposition of SN contributing to Li^+ conduction through the formation of Li_3N, the C 1s spectrum is predominantly comprised of a peak at 284.8 eV, attributed to the C-C/C-H bonds of the polymer backbone, with minor contributions from ester and nitrile groups. Furthermore, the Cl 2p spectrum exhibits

dominant ClO_4^- peaks at approximately 208 eV and 209.5 eV, accompanied by a negligible Cl^- signal at around 198 eV. This observation unambiguously corroborates that the deep eutectic structure within the electrolyte effectively suppresses the reduction decomposition of perchlorate anions.

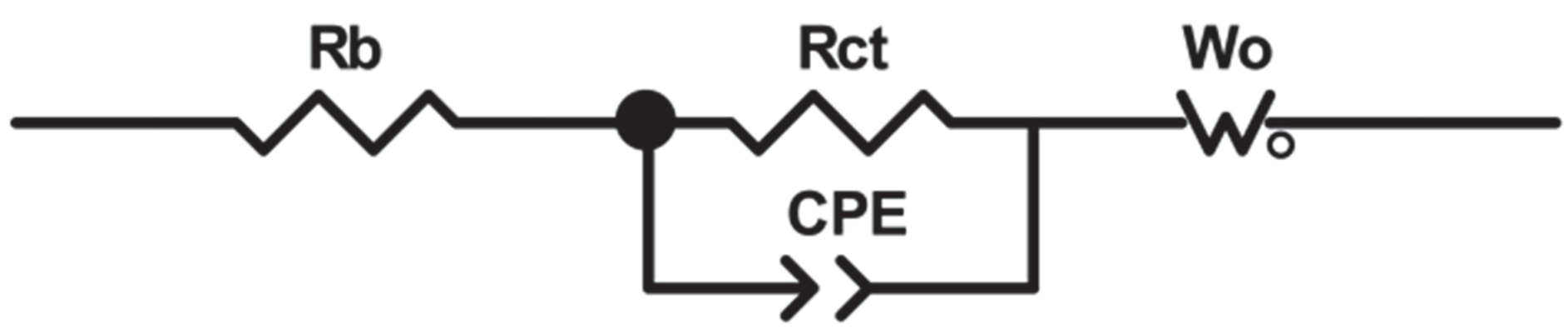

Figure S17. Equivalent circuit diagram for EIS fitting

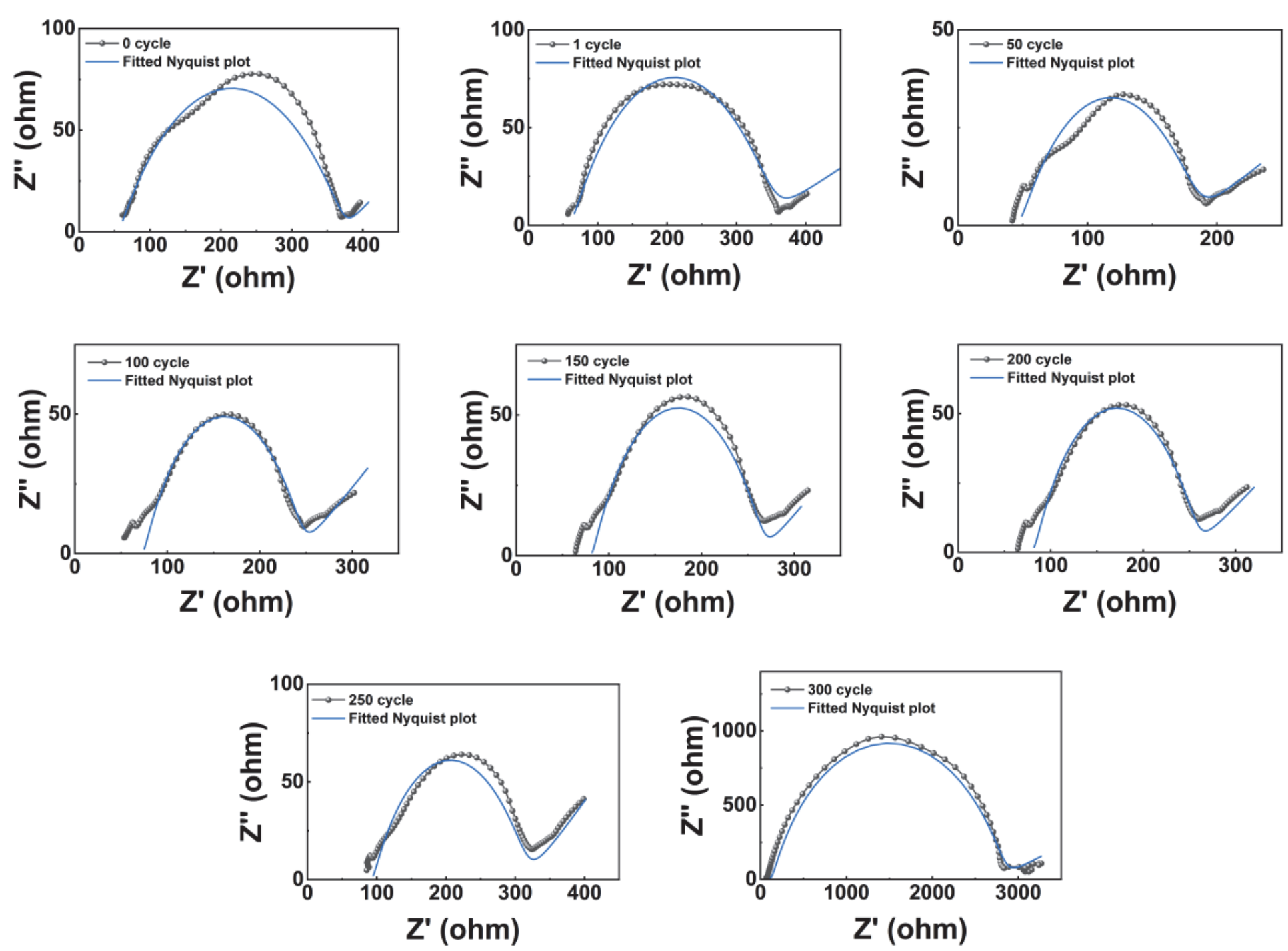

Figure S18. Fitted curves of EIS spectra at different cycle numbers

Table S3. Fitting data for EIS data

Cycle	R_b (Ω)	R_{ct} (Ω)	CPE-T ($S \cdot s^n$)	CPE-P	W_o-R ($\Omega/s^{0.5}$)	W_o-T ($\Omega/s^{0.5}$)	W_o-P ($\Omega/s^{0.5}$)
0	54.08	307.7	3.15×10^{-5}	0.539	29.6	45	0.117
1	47.97	267.5	1.12×10^{-5}	0.624	34.83	0.04	0.074
50	40.69	125.2	6.25×10^{-5}	0.579	26.6	1.01	0.082
100	70.74	162	2.40×10^{-5}	0.672	30.33	3.251	0.124
150	78.36	174.7	4.58×10^{-5}	0.669	35.33	50	0.120
200	75.57	163.8	2.26×10^{-5}	0.694	47.5	28.2	0.108
250	90.3	206.6	2.77×10^{-5}	0.654	75.7	88.42	0.147
300	65.11	2612	2.36×10^{-5}	0.766	210	0.867	0.092

Note 7: Nyquist plots exhibited semicircles in the medium-frequency region, corresponding to Li^+ charge transfer and diffusion. In the equivalent circuit used for EIS fitting, R_b, R_{ct}, CPE, and W represent the internal resistance, charge transfer resistance, constant phase elements, and Warburg impedance of the symmetric cells, respectively. The R_{ct} during cycling (160-200 Ω) is substantially reduced compared with the initial state (307 Ω), reflecting a marked decrease in the interfacial charge transfer barrier (all fitted parameters are summarized in Table S3 of the Supporting Information). This reduction is attributed to the strong affinity and low diffusion barrier of the poly($DEE_{0.8}$-MMA) electrolyte, facilitating homogeneous component distribution, the formation of interconnected networks, and shortened diffusion pathways, thereby enabling rapid ion transport. Consequently, a stable interfacial environment is established, enabling uniform Li plating, reducing diffusion distances, suppressing polarization, and extending the cycle life of the symmetric cells.

Collectively, these findings indicate that the poly($DEE_{0.8}$-MMA) electrolyte promotes uniform Li deposition on the anode, effectively suppresses side reactions and dendrite formation, and demonstrates excellent interfacial compatibility and electrochemical stability.

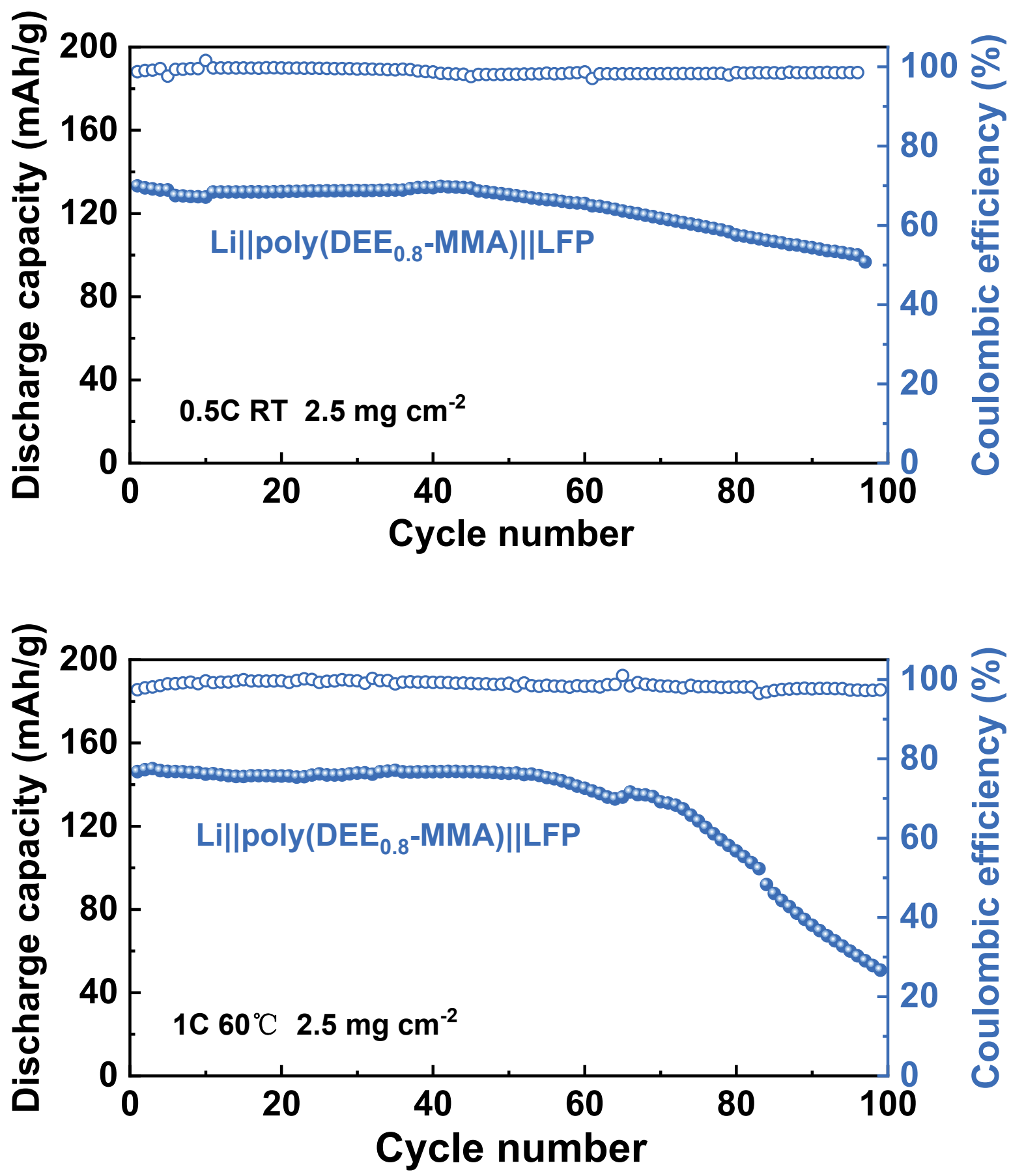

Figure S19. (a) Cycling performance of Li||poly($DEE_{0.8}$-MMA)||LFP cells at RT and 0.5 C; (b) Cycling performance of Li||poly($DEE_{0.8}$-MMA)||LFP cells at 60 ºC and 1 C.

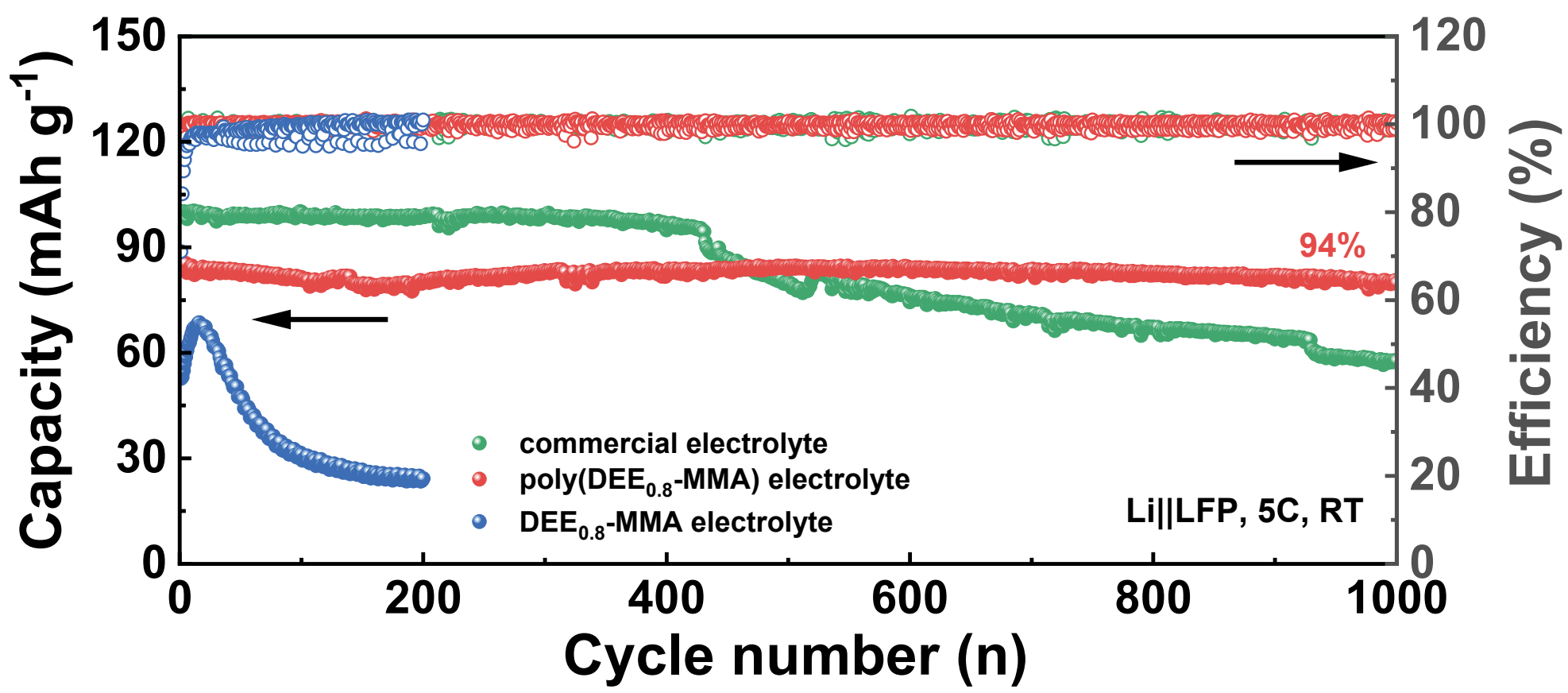

Figure S20. Cycling performance and Coulombic efficiency of Li| poly($DEE_{0.8}$-MMA)|LFP, Li|$DEE_{0.8}$-MMA|LFP, and Li|commercial electrolyte|LFP cells at 5 C.

Table S4. Comparison of cell performance with published papers

Electrolytes		state	Electrodes	Rate/cycle number/capacity retention	Ref.
Salt	Polymer matrix				
$LiClO_4$	poly(DEE-MMA)	solid	$LiFePO_4$||Li	1C/120 cycle/80%<br>5C/1000 cycle/94%	This work
$LiClO_4$	poly(MMA)	solid	$LiFePO_4$||Li	1C/50 cycle/20%	
LiTFSI	MPEGA-AM	solid	$LiFePO_4$||Li	0.45C/250 cycle/83%	[7]
LiTFSI	PVDF-PEO	solid	$LiFePO_4$||Li	0.3C/300 cycle/94.1%	[8]
LiTFSI	PEO	quasi-solid	$LiFePO_4$||Li	0.1C/100 cycle/87.9% (60 ºC)	[9]
LiTFSI	PDMAA	solid	$LiFePO_4$||Li	0.5C/300 cycle/98%	[10]
LiTFSI	UpyMA-PETEA	solid	$LiMn_2O_4$||Li	0.1C/200 cycle/86%	[11]
$LiClO_4$	PAN/TPU	solid	$LiFePO_4$||Li	0.12C/100 cycle/98%	[12]
LiTFSI	ETPTA+3F-NMA	solid	$LiFePO_4$||Li	5C/1000 cycle/77.4%	[13]
LiTFSI	PAM+SN	solid	$LiFePO_4$||Li	1C/200 cycle/96.1%<br>5C/1000 cycle/77.6%	[1]

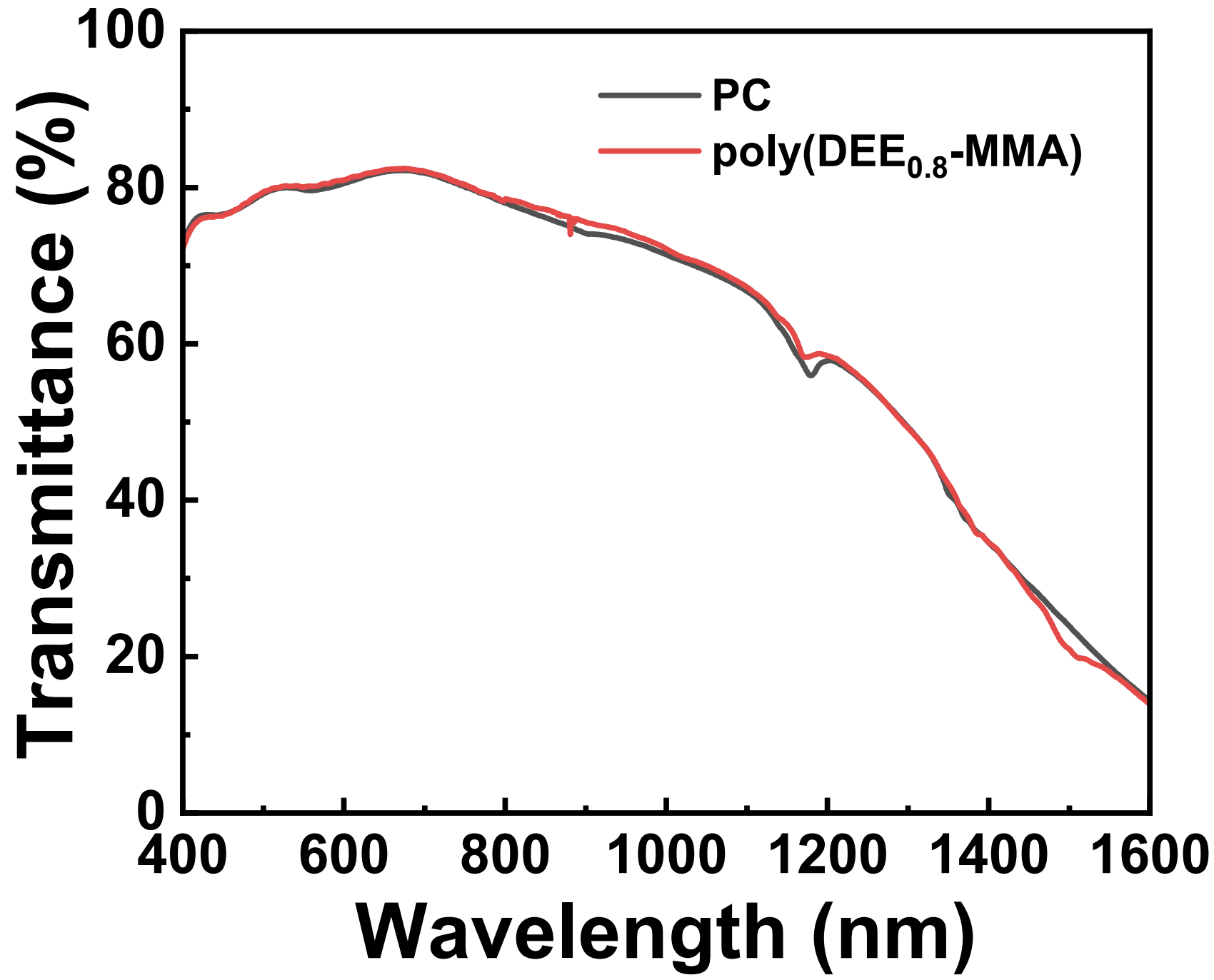

Figure S21. Transmittance spectra of Devices assembled based on poly($DEE_{0.8}$-MMA) SPE and PC-$LiClO_4$ solution.

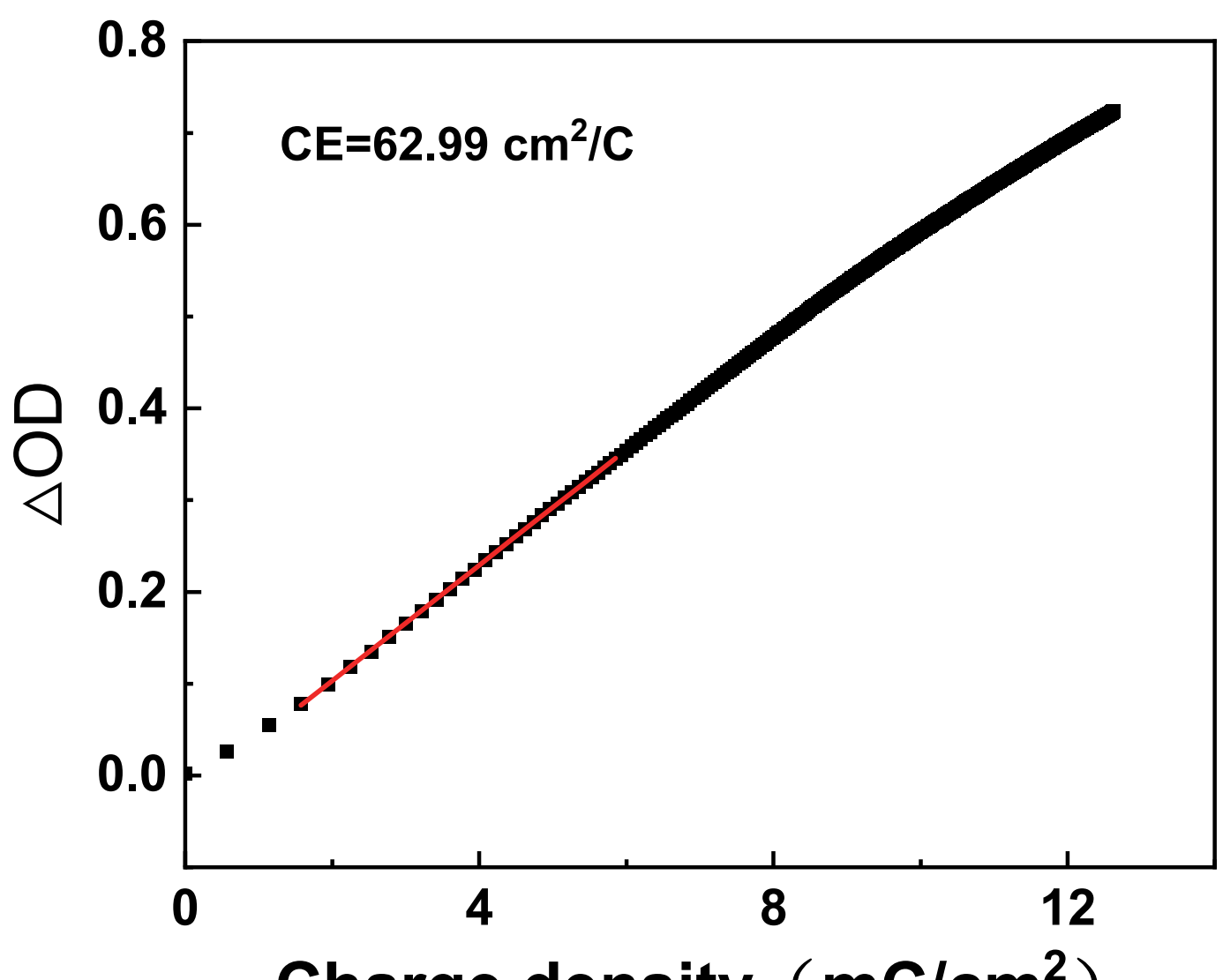

Figure S22. Plot of optical density (ΔOD) at 633 nm vs. the charge density of the semi-ECD.

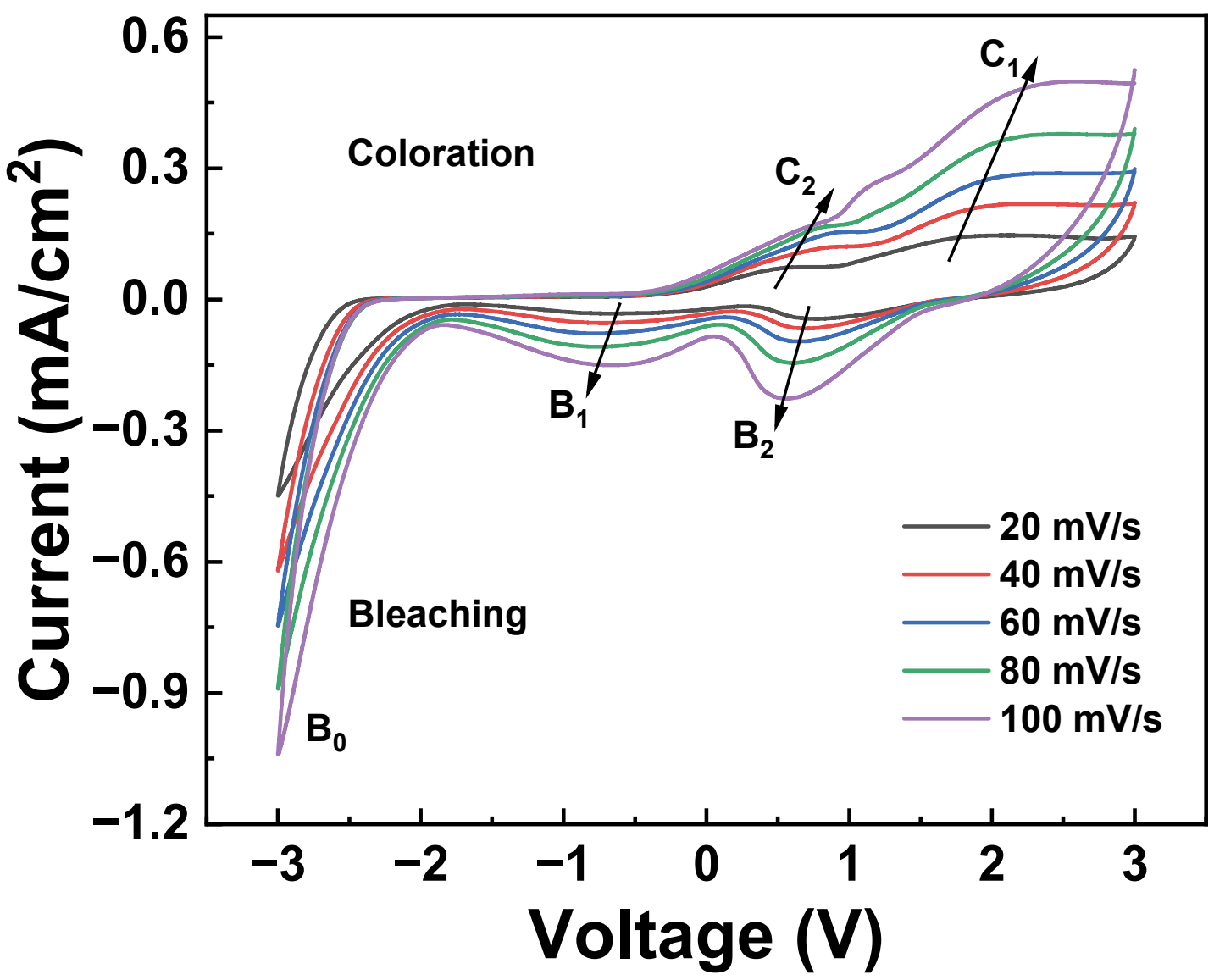

Figure S23. CV curves of FTO/PB/poly(DEE$_{0.8}$-MMA)/FTO ECD at various scan rates.

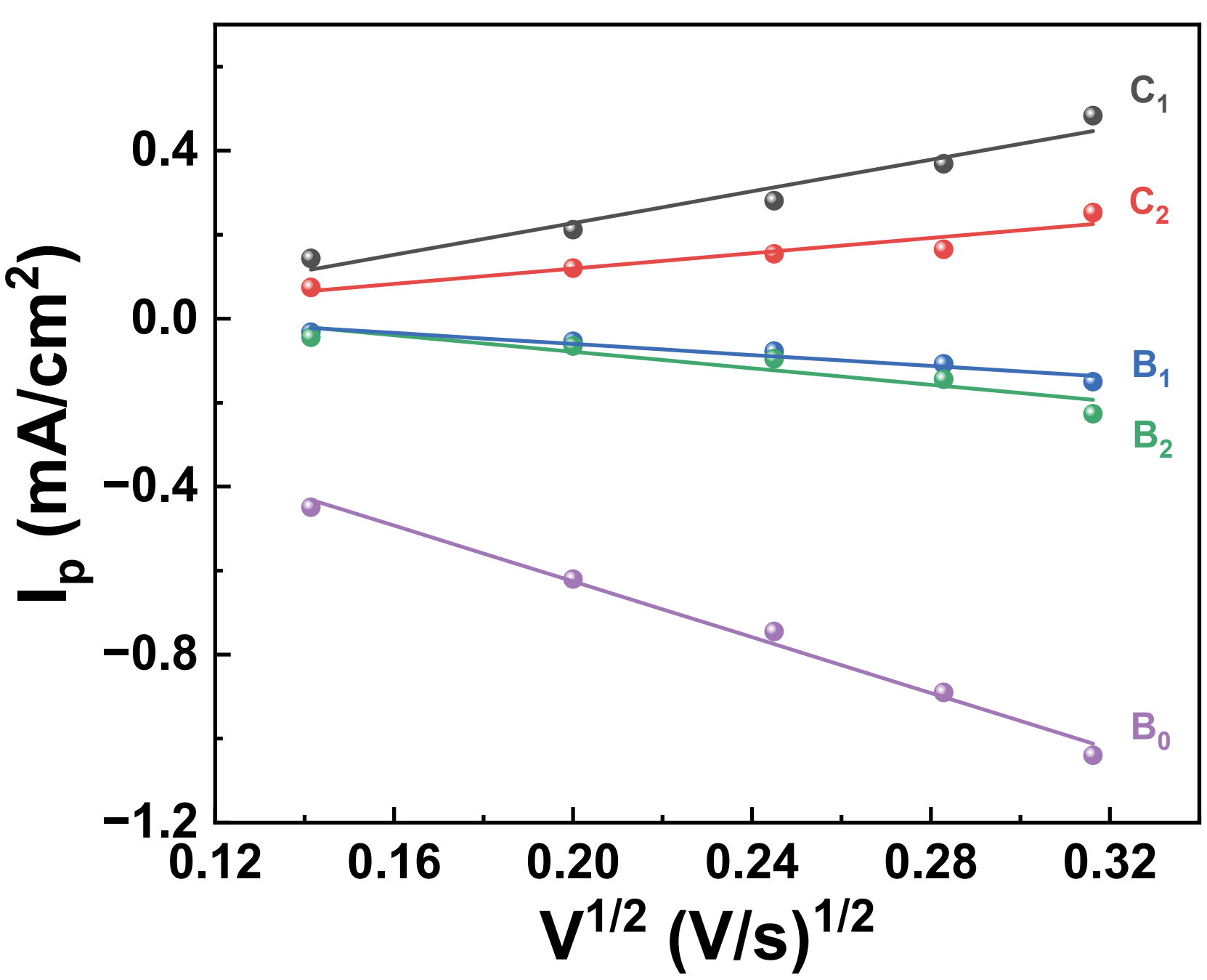

Figure S24. Coloration/bleaching peak current density as a function of the square root of the scanning rate.

Note 8: The effective diffusion coefficient can be calculated using the Randles-Sevcik equation as follows:

$$I_p = 2.69\times10^5 n^{\frac{3}{2}} A D_{Li^+}^{\frac{1}{2}} v^{\frac{1}{2}} C_{Li^+} \qquad \text{Equation S3}$$

where Ip is the peak current, n is the number of electrons in the reaction (n = 1), A is the area of electrodes in the electrolyte ($A \approx 6$ cm^2), D_{Li+} is the effective diffusion coefficient of Li^+ ions, ν is the scan rate, and C_{Li+} is the concentration of lithium ions (C_{Li^+}=1.5 × 10^{-3} mol mL^{-1}).

Table S5. Diffusion coefficients

Peak	Diffusion number (cm^2/s)
C_1	4.92 × 10^{-10}
C_2	1.14 × 10^{-10}
B_0	1.52 × 10^{-9}
B_1	5.87 × 10^{-11}
B_2	1.34 × 10^{-10}

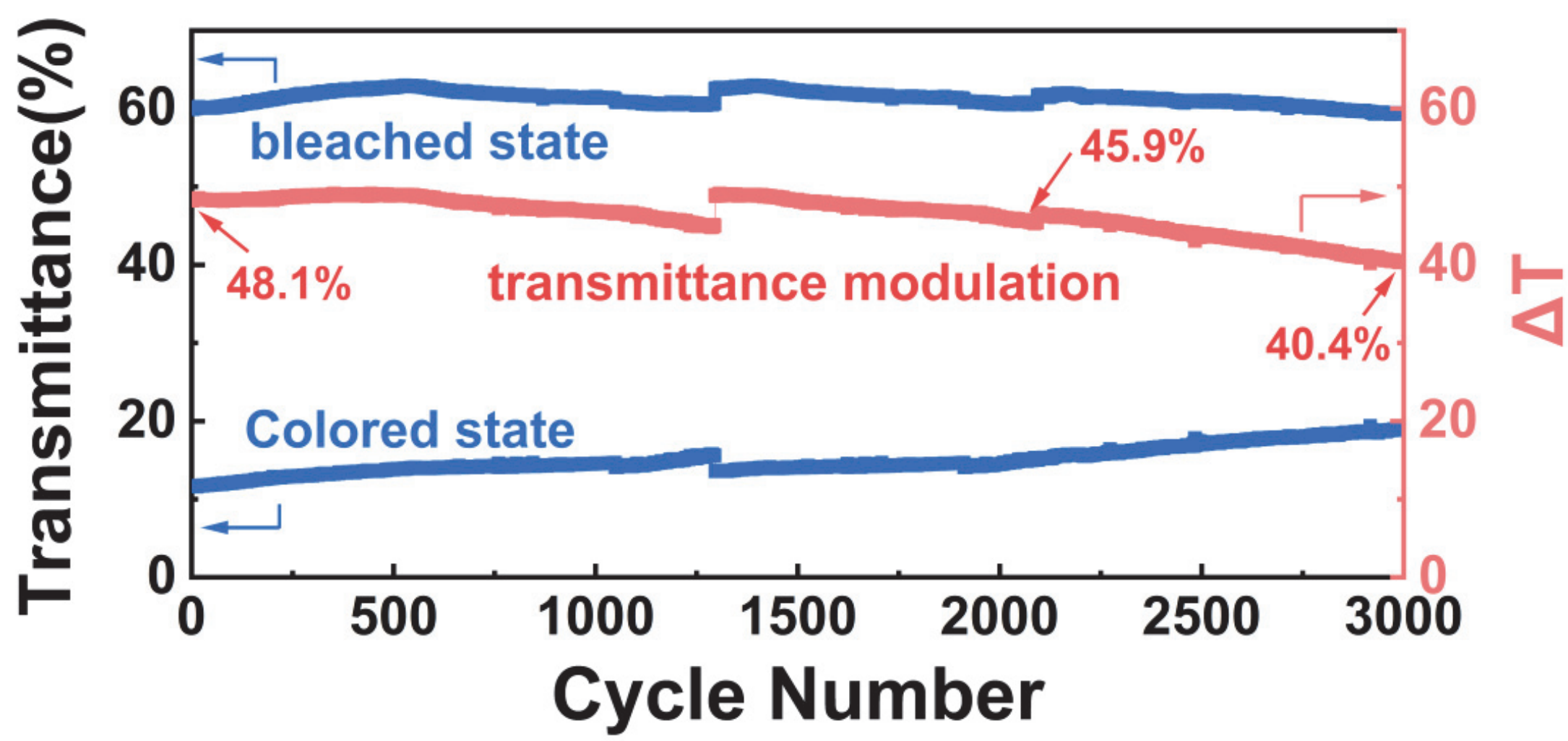

Figure S25. Transmittance at 633 nm of ECD over 3000 cycles (voltage range of -3.0 to 1.5 V).

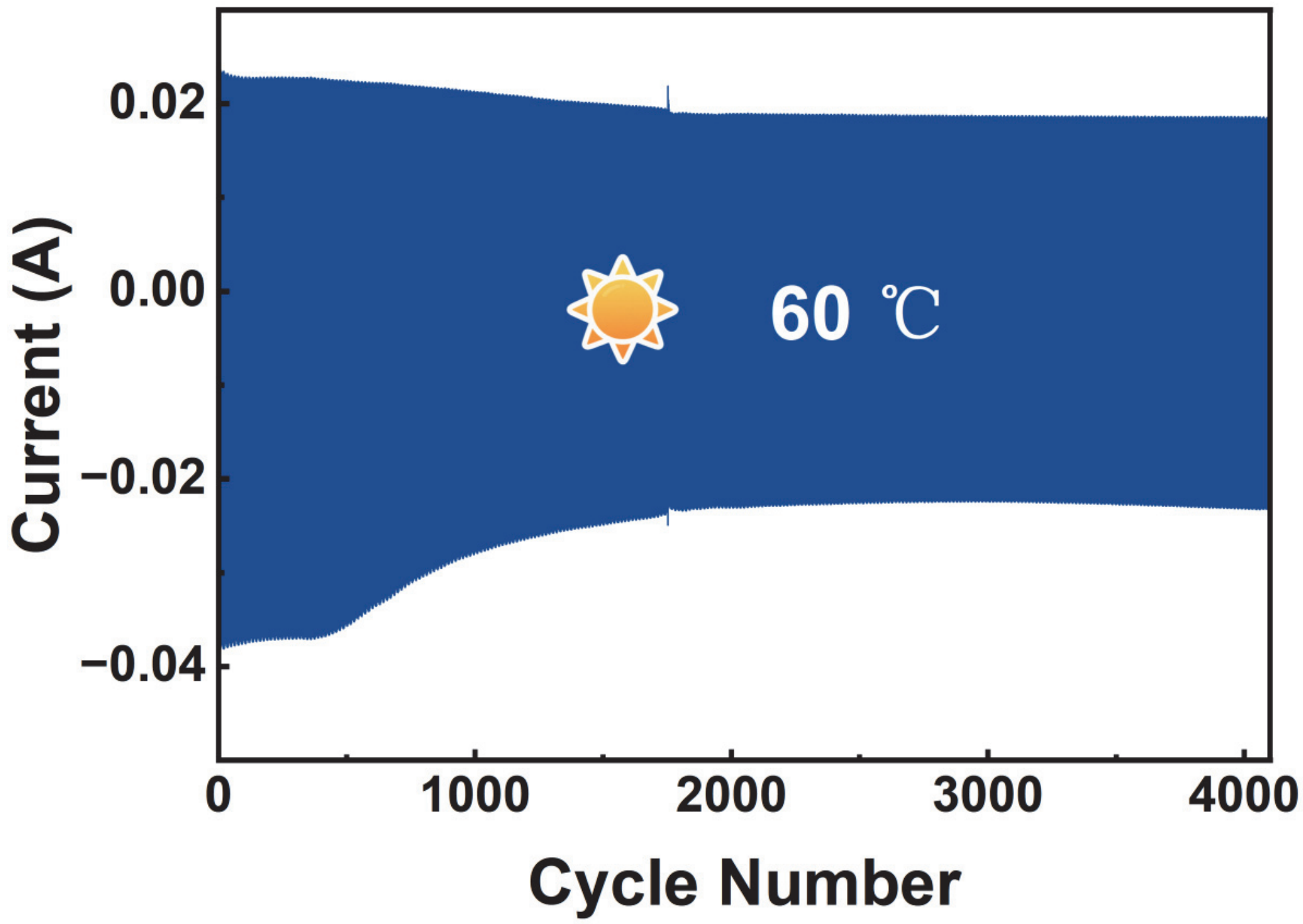

Figure S26. Time-current profiles corresponding to the FTO/PB/poly($DEE_{0.8}$-MMA)/FTO ECD recorded at 60 °C.

Table S6 The performance of ECDs utilizing polymer electrolytes

Electrolytes in the ECD	EC layer	Conductivity ($ms\cdot cm^{-1}$)	EC performance	Ref.
**P(DEE-MMA)+LiClO4**	PB	0.943	ΔT%=50.3%;<br>T_b/T_c=21.1/14.7 s;<br>CE=62.99 cm^2/C;<br>81% after 2000 cycles；<br>4000 cycles at 60 °C	This work
**1 M $LiClO_4$/PC**	$W_{18}O_{49}$/PB	-	ΔT%=71.2%;<br>T_b/T_c=4.2/2.4 s;<br>70% after 100 cycles；	[14]
**PVDF-HFP+ILs+LiTFSI**	PB	-	ΔT%=85%;<br>Tb/Tc=6.8/1.7 s;<br>CE=184.16 cm2/C;<br>90% after 1500 cycles；	[15]
**PVA+SN+H_3PO_4+LiTFSI**	WO_3	0.34	ΔT%=41%;<br>T_b/T_c=17/43 s;<br>CE=70.12 cm^2/C;<br>61% after 300 cycles；	[16]
**EAA+gelatin+$LiClO_4$**	WO_3	0.004	CE=60.38 cm^2/C;<br>T_b/T_c=50/10 s;	[17]
**PMMA+$LiClO_4$**	WO_3	0.58	ΔT%=60.92%;<br>T_b/T_c=23.8/38.1 s;<br>CE=53.77 cm^2/C;<br>87% after 500 cycles；	[18]
**P(DEEA-co-IOBA)+LiTFSI**	WO_3/NiO	0.63	ΔT%=45%;<br>T_b/T_c =8.4/11.4 s；<br>CE=150 cm^2/C;<br>91.2% after 250 cycles；	[19]
**P(MMA-BMA-HEMA)+$LiClO_4$ (PC)**	WO_3	4.02	ΔT%=34.5%；<br>90 % after 2840 cycles	[20]

Reference

[1] X. Liang, C. Liu, S. Liao, S.X. Yao, M. He, Polymerizable Deep Eutectic Solvent-Based Polymer Electrolyte for Advanced Dendrite-Free, High-Rate, and Long-Life Li Metal Batteries, ACS Appl. Mater. Interfaces 16 (2024) 4661–4670. https://doi.org/10.1021/acsami.3c15889.

[2] F. Zhao, Y. Zeng, Z. Cheng, G. Shi, Q. Liu, Y. Liu, G. Han, Cathode/Anode electrodes for large-area bifunctional electrochemical devices prepared by a novel Na3Cit-assisted chemical deposition method, Chemical Engineering Journal 485 (2024) 149350. https://doi.org/10.1016/j.cej.2024.149350.

[3] F. Neese, The ORCA program system, WIREs Computational Molecular Science 2 (2012) 73–78. https://doi.org/10.1002/wcms.81.

[4] T. Lu, F. Chen, Multiwfn: A multifunctional wavefunction analyzer, Journal of Computational Chemistry 33 (2012) 580–592. https://doi.org/10.1002/jcc.22885.

[5] X. Wu, Y. Zhang, S. Peng, An ambient-temperature superionic conductive, electrochemically stable, plastic cross-linked polymer electrolyte for lithium metal battery, Journal of Applied Polymer Science 141 (2024) e55234. https://doi.org/10.1002/app.55234.

[6] L. Wang, Y. He, H.L. Xin, Transition from Vogel-Fulcher-Tammann to Arrhenius Ion-Conducting Behavior in Poly(Ethyl Acrylate)-Based Solid Polymer Electrolytes via Succinonitrile Plasticizer Addition, J. Electrochem. Soc. 170 (2023) 090525. https://doi.org/10.1149/1945-7111/acf881.

[7] S. Yun, S. Kim, P.L. Handayani, S. Jung, J.H. Park, U.H. Choi, Dynamic Networks via Polymerizable Deep Eutectic Monomers for Uniform Li^+ Transport at Interfaces in Lithium Metal Batteries, Advanced Functional Materials 35 (39) 2500232. https://doi.org/10.1002/adfm.202500232.

[8] S. Yang, X. He, T. Hu, Y. He, S. Lv, Z. Ji, Z. Zhu, X. Fu, W. Yang, Y. Wang, A Supertough, Nonflammable, Biomimetic Gel with Neuron-Like Nanoskeleton for Puncture-Tolerant Safe Lithium Metal Batteries, Advanced Functional Materials 33 (2023) 2304727. https://doi.org/10.1002/adfm.202304727.

[9] Q. Liu, Y. Dan, Y. Niu, Y. Lv, G. Li, A Highly Compatible Deep Eutectic Solvent-Based Poly(ethylene) Oxide Polymer Electrolyte to Enable the Stable Operation of 4.5 V Lithium Metal Batteries, Small 21 (2025) 2408944. https://doi.org/10.1002/smll.202408944.

[10] long Wan, H. Nie, Q. Yu, Design of ultrafast lithium ion channel for solid-state lithium metal batteries by in-situ polymerization induced phase separation, Chemical Engineering Journal 513 (2025) 162810. https://doi.org/10.1016/j.cej.2025.162810.

[11] P. Jaumaux, Q. Liu, D. Zhou, X. Xu, T. Wang, Y. Wang, F. Kang, B. Li, G. Wang, Deep-Eutectic-Solvent-Based Self-Healing Polymer Electrolyte for Safe and Long-Life Lithium-Metal Batteries, Angewandte Chemie International Edition 59 (2020) 9134–9142. https://doi.org/10.1002/anie.202001793.

[12] X. Lu, J. Luo, L. Lan, Y. Wang, X. Liang, J. Li, A. Fu, Composite Polymer Electrolyte Based on PAN/TPU for Lithium-Ion Batteries Operating at Room Temperature, Polymers 16 (2024) 3280. https://doi.org/10.3390/polym16233280.

[13] Y. Lei, B. Chen, H. Tian, Z. Zeng, S. Fang, D. Li, L. Ci, Q. Yuan, ETPTA polymer network confined amide-based eutectic electrolyte for safe and long- life lithium metal battery, Chemical Engineering Journal 502 (2024) 158180. https://doi.org/10.1016/j.cej.2024.158180.

[14] Z. Wang, Q. Zhang, S. Cong, Z. Chen, J. Zhao, M. Yang, Z. Zheng, S. Zeng, X. Yang, F. Geng, Z. Zhao, Using Intrinsic Intracrystalline Tunnels for Near-Infrared and Visible-Light Selective Electrochromic Modulation, Advanced Optical Materials 5 (2017) 1700194. https://doi.org/10.1002/adom.201700194.

[15] H. Gong, A. Li, G. Fu, M. Zhang, Z. Zheng, Q. Zhang, K. Zhou, J. Liu, H. Wang, Ultrathin flexible electrochromic devices enabled by highly transparent ion-conducting films, J. Mater. Chem. A 11 (2023) 8939–8949. https://doi.org/10.1039/D2TA09807E.

[16] H. Xu, I. Haider, Y. Zheng, W. Li, S. Zhuiykov, Y. Cui, Towards the

Solid-State electrochromic devices: Platform based on transparent and flexible solid polymer electrolyte, Chemical Engineering Journal 508 (2025) 161116. https://doi.org/10.1016/j.cej.2025.161116.

[17] J. Wootthikanokkhan, P. Jaruphan, M.H. Azarian, J. Yosthisud, Effects of ethylene-acrylic acid ionomer on thermomechanical and electrochromic properties of electrochromic devices using gelatin-based electrolytes, Journal of Applied Polymer Science 137 (2020) 49362. https://doi.org/10.1002/app.49362.

[18] T. Chen, F. Zhao, L. Wang, S. Ma, G. Shi, Q. Liu, Y. Liu, G. Han, High-performance PMMA based solvent-free solid transparent polymer electrolyte modified by succinonitrile for electrochromic devices, Solar Energy Materials and Solar Cells 285 (2025) 113538. https://doi.org/10.1016/j.solmat.2025.113538.

[19] P. Sun, J. Chen, Y. Li, X. Tang, H. Sun, G. Song, X. Mu, T. Zhang, X. Zha, F. Li, Y. Gao, S. Cong, Z. Zhao, Deep eutectic solvent-based gel electrolytes for flexible electrochromic devices with excellent high/low temperature durability, InfoMat 5 (2023) e12363. https://doi.org/10.1002/inf2.12363.

[20] Z. Zhou, Y. Tang, F. Zhao, G. Li, G. Xu, Y. Liu, G. Han, Transparent succinonitrile-modified polyacrylate gel polymer electrolyte for solid electrochromic devices, Chemical Engineering Journal 481 (2024) 148724. https://doi.org/10.1016/j.cej.2024.148724.
